# Development and validation of a novel predictive model for dementia risk in middle-aged and elderly depression individuals: a large and longitudinal machine learning cohort study

**DOI:** 10.1186/s13195-025-01750-6

**Published:** 2025-05-13

**Authors:** Xuan Xiao, Yihui Li, Qiaoboyang Wu, Xinting Liu, Xu Cao, Maiping Li, Jianjing Liu, Lianggeng Gong, Xi-jian Dai

**Affiliations:** 1 Department of Radiology, The Second Affiliated Hospital, Jiangxi Medical College, Nanchang University, Minde Road No. 1, Nanchang, Jiangxi Province, 330006 China; 2 Jiangxi Provincial Key Laboratory of Intelligent Medical Imaging, Nanchang, 330006 China

**Keywords:** Dementia, Depression, Machine learning, Risk prediction, UK Biobank, DRP-Depression, Web application

## Abstract

**Background:**

Depression serves as a prodromal symptom of dementia, and individuals with depression exhibit a significantly higher risk of developing dementia. The aim of this study is to develop and validate a novel dementia risk prediction tool among middle-aged and elderly individuals with depression based on machine learning algorithms.

**Methods:**

This study included 31,587 middle-aged and elderly individuals with depression who did not have a diagnosis of dementia at baseline from a large UK population-based prospective cohort. A rigorous variable selection strategy was employed to identify risk and protective factors of dementia from an initial pool of 190 candidate variables, ultimately retaining 27 variables. Eight distinct data analysis strategies were utilized to develop and validate the dementia risk prediction model. The DeLong's test was applied to compare the statistical differences between different models.

**Results:**

During a median follow-up of 7.98 years, 896 incident dementia cases were identified among study participants. In model development employing an 8:2 data split (fivefold cross-validation for training), the Adaboost classifier achieved the optimal performance (AUC 0.861 ± 0.003), followed by XGBoost (AUC 0.839 ± 0.005) and CatBoost (AUC 0.828 ± 0.007) classifiers. To facilitate community generalization and clinical applicability, we develop a simplified model through a forward feature subset selection algorithm, retaining 12 variables. The simplified model maintained robust performance, with AdaBoost achieving the highest discriminative ability (AUC 0.859 ± 0.002), followed by XGBoost (AUC 0.835 ± 0.001) and CatBoost (AUC 0.821 ± 0.005). The DeLong's test revealed no statistically significant difference in AUC values between models using 12 and 27 variables (*p* = 0.278). For practical implementation, we deployed the optimal model to a web application for visualization and dementia risk assessment, named DRP-Depression.

**Conclusions:**

We developed a practical and easy-to-promote risk prediction model based on machine learning algorithms, and deployed it to a web application to provide a new and convenient tool for dementia risk prediction in the middle-aged and elderly individuals with depression.

**Supplementary Information:**

The online version contains supplementary material available at 10.1186/s13195-025-01750-6.

## Background

Dementia, ranked as the seventh leading cause of mortality globally, afflicts an estimated 55 million adults worldwide, with epidemiological projections indicating a doubling of prevalence every two decades [1, 2]. Despite significant research investments in elucidating etiological pathways and developing preventive strategies, no disease-modifying therapies have achieved clinical validation to date. This therapeutic impasse underscores the paramount importance of both the identification of modifiable risk factors and the implementation of early interventions for mitigating disease incidence and attenuating pathological progression in pre-symptomatic stages [3, 4].

Depressive disorders affect over 300 million people, equivalent to 4**.**4% of the world population, and have become the leading cause of global disease burden [5]. Evidence reveals a significantly higher risk of developing dementia in individuals with depression compared to those without depression, independent of depression onset chronology [6]. This robust association persists across longitudinal cohort studies [7–9], necessitating enhanced clinical vigilance for neurocognitive decline in this population.

Mechanistically, genome-wide association studies identify the CALHM2 V136G mutation as a shared genetic vulnerability, where impaired astrocytic ATP release capacity mediates both depressive symptomatology and Alzheimer-type neurodegeneration [10]. These findings corroborate the hypothesis of overlapping neurodegenerative pathways between the two disorders. Dementia-related pathological changes may be present in patients'decades before clinical manifestation appear, with depression frequently presenting as a prodromal symptom preceding overt cognitive decline by decades [11–14]. Therapeutic challenges emerge in geriatric populations. The elderly are more sensitive to antidepressants and have more side effects, and some elderly patients with executive function deficits show do not respond adequately to antidepressant therapy [15]. Identifying potential risk factors and implementing timely interventions could help prevent the onset of dementia and improve the outcome of dementia patients [16, 17]. Therefore, integrating depression-specific dementia risk prediction algorithms into routine psychiatric evaluations could optimize precision prevention frameworks for high-risk populations.

Various clinical decision-support tools have been developed to help clinicians to stratify dementia risk in clinical practice. Notable examples include: (1) The Cardiovascular Risk Factors, Aging, and Incidence of Dementia (CAIDE) Risk Score is a tool that estimates the risk of developing dementia in 20 years based on several vascular risk factors (e.g., education level, stroke, total cholesterol, body mass index) [18]; (2) The Brief Dementia Screening Index (BDSI) is a primary care-optimized instrument for dementia that can be used by non-specialists [19]; (3) The UK Biobank dementia risk prediction (UKB-DRP) model developed by You et al., employed machine learning methods to estimate 5-, 10-, and longer-periods risk of dementia in the general population [20]. Nevertheless, these models exhibit critical limitations for routine implementation, particularly their reliance on biomarkers with restricted clinical accessibility (e.g., CAIDE’s total cholesterol, UKB-DRP's ApoE genotype) that hinder cost-effective mass screening. Our research team recently addressed these limitations by creating a point-based risk score prediction model to evaluate the 5-, 9-, and 13-year risk of dementia, which demonstrated superior predictive performance (AUC = 0.87) using exclusively clinically accessible variables [21]. However, existing models are all developed based on general population. To our knowledge, reports of developing clinical decision support tools to predict dementia risk specifically tailored for depression population have not been found, leaving a critical evidence gap regarding dementia risk stratification in depression comorbidity contexts.

In the present large-scale longitudinal diagnostic study, we employed machine learning techniques to develop a simple and convenient dementia risk prediction tool specifically tailored for middle-aged and elderly depression population. This study is analysed based on data from the comprehensive prospective UK Biobank cohort. We implemented a rigorous feature selection strategy to obtain optimal variables associated with dementia that were readily available. To facilitate community generalization and clinical applicability, we deploy a simplified and optimal prediction model to an interoperable Web-based interface application that can automates risk stratification using routinely collected clinical parameters. Our study provides healthcare practitioners with a novel digital solution for point-of-care dementia risk stratification in depression population.

## Methods

### Study participants

This study holds de-identified data of 502,386 participants aged 37–73 enrolled in the UK Biobank between March 13, 2006, and October 1, 2010. All participants who completed the baseline assessment were included from 22 recruitment centers across England, Scotland, and Wales.Among the de-identified data of 502,386 participants, 31,587 (6.29%) case were identified as having depression. Comprehensive data collection included structured interviews, self-reported questionnaires, and standardized assessments capturing demographic characteristics, lifestyle factors, health status, physical measurements, biospecimen data, imaging data, and phenotypic characteristics. Ethical approval was granted by the North West Multi-Center Research Ethics Committee (REC reference: 16/NW/0274) and the Second Affiliated Hospital of Nanchang University. All enrolled individuals provided written informed consent prior to participation. This study followed the Transparent Reporting of a Multivariable Prediction Model for Individual Prognosis or Diagnosis (TRIPOD) reporting guideline and was conducted from March 31 to August 31, 2023.

### Primary outcome

The primary startpoint of this study was the first documented occurrence of depression, and the primary endpoint was the first documented occurrence of dementia. For those who were not identified as incident dementia cases during follow-up, the endpoint was March 31, 2023 or the date of death if the death data occurred earlier. Information on disease diagnosis and date of diagnosis were coded according to the International Statistical Classification of Diseases, 10 th revision (ICD-10) terms from UK Biobank data field 41270 and 41280, respectively (dementia, Field ID F01–F05 and G30; depression, F32–F33, F41**.**2, and T43**.**0-T43**.**2; Supplementary eTable 1). The primary outcome of dementia included Alzheimer's disease, vascular dementia, dementia unspecified organic amnestic syndrome, and dementia in other disorders of other classifications.

### Candidate variables

This study initially included all clinically relevant variables obtained at baseline assessments. Subsequently, following multidisciplinary clinical review, procedural metric variables that were clinical meaningless (e.g., biospecimen processing metrics, diagnosis codes, measure device, IDs) and some information variables used to record time were excluded. Variable selection was guided by three principal considerations: (1) validated dementia risk factors from epidemiological studies [22]; (2) depression-related biomarkers [23]; and (3) clinical phenotypes demonstrating mechanistic relevance to neurodegeneration (e.g., sleep disorders, health maintenance behaviors). Subsequent categorization organized the final 190 variables into 9 clinically meaningful domains (Supplementary eTable 2), including the demographic characteristics (*n* = 5), lifestyle and environmental exposures (*n* = 51), sleep phenotypes (*n* = 8), socioeconomic status (*n* = 10), medications and medical history (*n* = 53), laboratory tests (*n* = 23), physical measures (*n* = 10), social and psychological factors (*n* = 29), and a self-generated variable (*n* = 1).

### Variable selection

A rigorous multi-stage selection framework was implemented to optimize feature-outcome associations. The variable filtration protocol comprised four sequential phases: (1) Statistical filtering employing Chi-square test for categorical variables and Mann-Whitney-Wilcoxon test for continuous variables, eliminating non-significant 87 variables (*p* > 0.05); (2) Completeness evaluation excluding 54 variables with > 5% missing data; (3) Clinical feasibility assessment removing 20 variables requiring specialized collection protocols or not easily available; (4) multicollinearity association removing 2 variables with variance inflation factor (VIF) values > 5 (Supplementary eFigure 1). The resultant 27 variables were used to develop dementia risk prediction model.

### Forward feature subset selection

Forward feature subset selection is a computationally efficient stepwise algorithm balancing model parsimony and predictive power [24]. Our implementation protocol comprised: 1) Initialization with null feature space; 2) Iterative AUC maximization through fivefold cross-validation; 3) Greedy incorporation of features $f_{j}$, yielding maximal incremental discrimination;$f_{j} = \arg \max_{{f_{i} }} (AUC(S \cup \{ f_{i} \} ) - AUC(S))$; 4) Termination upon three consecutive iterations with < 1% AUC improvement; 5) Final validation using independent test cohorts.

### Model development and validation framework

**Fig. 1:**
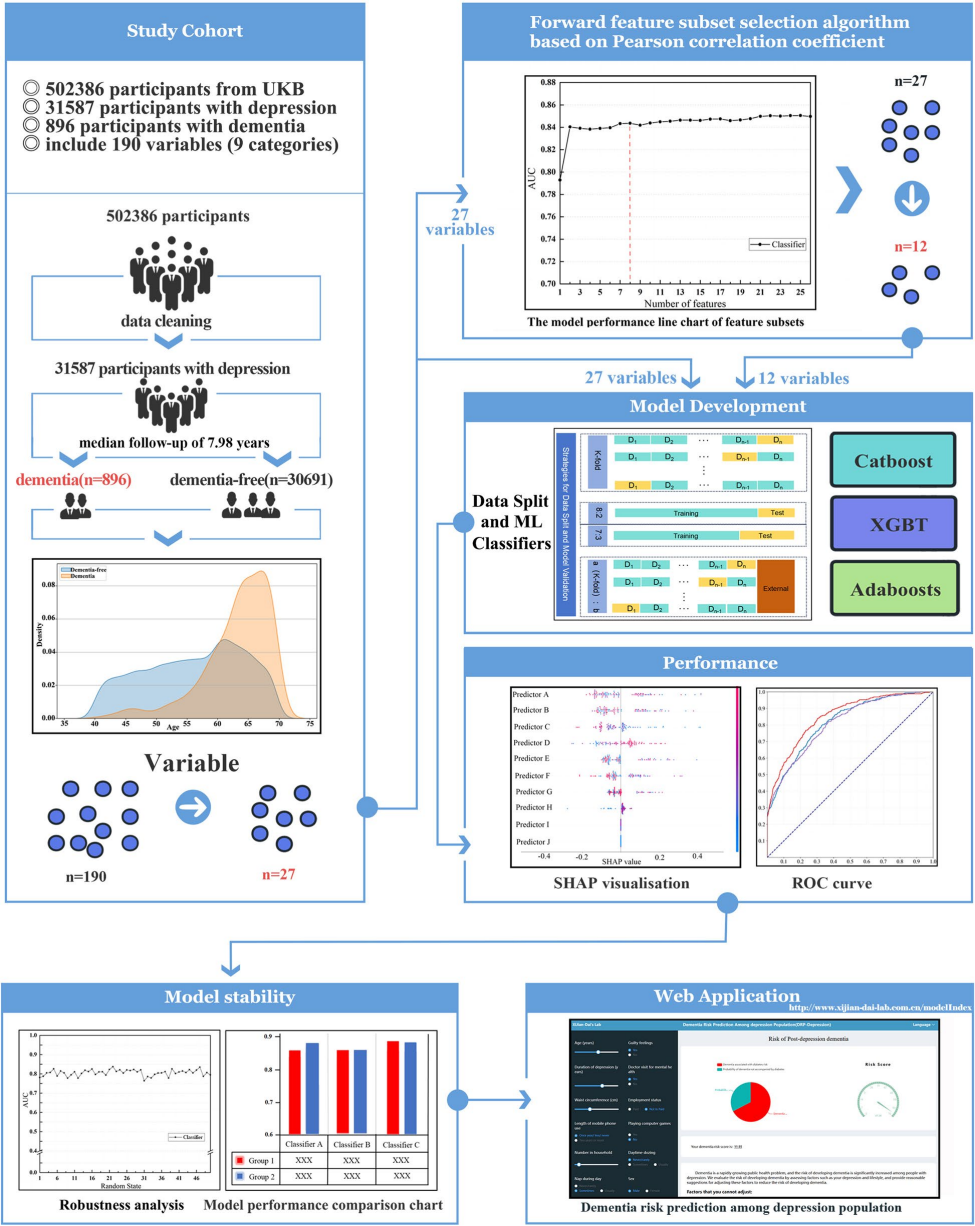
Experimental flow chart. The panels exhibit the development pipeline of the machine learning model. The diagram shows the key steps of model development, as well as sample diagrams of performance evaluation and web application process

A multi-stratum validation framework was implemented on the 31,587 depressed middle-aged and elderly participants. Eight distinct validation paradigms were systematically evaluated (Fig. 1).

1. Full cross-validation schem

**Study 1:** 10-fold cross-validation design A 10-fold cross-validation was used for model development and validation.

**Study 2**: 5-fold cross-validation design A 5-fold cross-validation was used for model development and validation.

**Study 3:** Data splitting by 8:2 We split the 31,587 depressed participants into training and testing datasets by a ratio of 8:2 to perform internal validation. To ensure the balanced distribution of patients with dementia between the training and testing datasets, we randomly split 80% of participants with dementia and 80% of those without dementia in the training dataset (9,226 men and 16,043 women), and 20% of participants with dementia and 20% of those without dementia in the testing dataset (2,307 men and 4,011 women).

2) Hybrid validation schemes

**Study 4:** Data splitting by 8:2 and 5-fold cross-validation

Similar to Study 3, the datasets were split into 8:2 for training and testing datasets, respectively. A 5-fold cross-validation was performed in the training datasets.

**Study 5:** Data splitting by 8:2, 5-fold cross-validation, and transfer learning model

Similar to Study 4, 5-fold cross-validation was used to develop training model in the 80% of depressed participants, the training model was then transferred to the 20% of depressed participants.

**Study 6:** Data splitting by 7:3

Similar to Study 3, but the dataset was split by 7:3.

**Study 7:** Data splitting by 7:3 and 5-fold cross-validation

Similar to Study 4, but the dataset was split by 7:3.

**Study 8:** Data splitting by 7:3, 5-fold cross-validation, and transfer learning model

Similar to Study 5, but the dataset was split by 7:3.

### Machine learning classifiers

In this study, three distinct machine learning classifiers were used to assess the performance of dementia prediction models:, Categorical Boosting (CatBoost) [25], Adaptive Boosting (AdaBoost) [26], and eXtreme Gradient Boosting (XGBoost) [27]. CatBoost is a gradient boosting-based classifier specifically optimized for classification features, while AdaBoost and XGBoost are ensemble learning methods that iteratively improve classification accuracy through weighted error correction. We dichotomized these models to stratify depressed participants into two prognostic categories: class 0 (expected to remain dementia-free) and class 1 (expected to develop incident dementia). Model performance was rigorously evaluated using receiver operating characteristic (ROC) curve analysis, with the area under the curve (AUC) serving as the primary evaluation metric. A comprehensive set of performance indicators was calculated, including classification accuracy, sensitivity, specificity, precision, and F1-score.

### Sensitivity analysis for temporal confounding

To address potential temporal bias in the depression-dementia association, we conducted sequential sensitivity analyses employing stratified exclusion criteria. The primary analysis excluded participants within 6 months post-diagnosis of depression (*n* = 109) to account for potential reverse causation, while the extended analysis removed those within 2 years (*n* = 1,423).. Following these temporal exclusion thresholds, we systematically re-evaluated model performance metrics (AUC, sensitivity, specificity) on the resultant restricted cohorts to assess the robustness of our predictive framework.

### Statistical analysis

Continuous variables are summarized using the mean ± standard deviation (SD). Categorical variables are presented as numbers (percentages). Group comparisons (dementia-free group vs dementia group) are conducted using Chi-square test for categorical variables and Mann-Whitney-Wilcoxon test for continuous variables. Missing data were imputed using the mode.

The DeLong's test [28] was used to compare the statistical differences in AUCs between the optimal model using 27-variable model and the simplified 12-variable model. We developed a webpage application for individual risk prediction based on the optimal model. Robustness analysis was used to evaluate the stability of the classification models. We used SHAP plots [29] to visualize how much each variable affects the target outcome.

To further characterize dementia risk associations, we implemented a multivariable Cox proportional hazard regression model, deriving adjusted hazard ratios (HRs) with 95% confidence intervals. All analyses were performed using IBM SPSS version 26**.**0 and Scikit-learn version 0**.**24**.**2 in Python version 3**.**6**.**8. Statistical significance was determined as *P* values < 0**.**05 using two-tailed hypothesis testing.

## Results

### Population characteristics

As shown in Supplementary eFigure 2, our initial cohort underwent rigorous exclusion criteria: 470,089 non-depressed participants and 710 participants whose diagnosis data of dementia predate that of depression were sequentially excluded. The remaining 31,587 depressed participants (mean age, 56**.**23 ± 8**.**10 years; 63**.**50% females) were included in the study. During a median follow-up of 7.98 years (interquartile range [IQR], 4.61–12.08), 896 (2**.**80%) incident dementia cases (mean age, 63**.**11 ± 5**.**51 years; 52**.**30% females) were identified (dementia group), and 30,691 (97**.**20%) participants (mean age, 56**.**03 ± 8**.**07 years; 63**.**80% females) remained dementia-free (dementia-free group).

Table 1 presents the demographic characteristics of the study population. The dementia cohort was significantly older at baseline compared to dementia-free counterparts (*p* < 0**.**001). Compared with the dementia-free group, the dementia group showed a higher prevalence rates of sleep problems, slow walking pace, obesity and high-frequency drinking, and a lower prevalence rates of employment status, visiting psychologist, and household members. Complete operational definitions and data acquisition protocols for all 27 candidate variables are shown in Supplementary eTable 3.

**Table 1:** The baseline characteristics of UK Biobank participants involved in the study

	Overall	Dementia group	Dementia-free group	
Characteristic	(*n*=31 587)	(*n*=896)	(*n*=30 691)	*p*-value
Age (mean±SD), y	56.23 ± 8.10	63.11 ± 5.51	56.03 ± 8.07	<.001
Sex				
Female	20 054 (63.50%)	469 (52.30%)	19 585 (63.80%)	<.001
Male	11 533 (36.50%)	427 (47.70%)	11 106 (36.20%)	
Employment status				
In paid	13 677 (43.30%)	155 (17.30%)	13 522 (44.06%)	<.001
Not in paid	17 470 (55.31%)	726 (81.03%)	16 744 (54.56%)	
Sleep duration				
7–8 h	17 045 (53.96%)	482 (53.79%)	16 563 (53.97%)	.001
≤6 h	9 689 (11.68%)	240 (26.79%)	9 449 (30.79%)	
≥9 h	4 288 (13.58%)	154 (17.19%)	4 134 (13.47%)	
Nap during day				
Never/rarely	14 144 (44.78%)	336 (37.50%)	13 808 (44.99%)	<.001
Sometimes	14 386 (45.54%)	421 (46.99%)	13 965 (45.50%)	
Usually	2 891 (9.15%)	134 (14.96%)	2 757 (8.98%)	
Sleeplessness				
Never	4 429 (14.02%)	156 (17.41%)	4 273 (13.92%)	.003
Sometimes/usually	27 026 (85.56%)	736 (82.14%)	26 290 (85.66%)	
Daytime dozing				
Never	21 036 (66.60%)	528 (58.93%)	20 508 (66.82%)	<.001
Sometimes	8 252 (26.12%)	273 (30.47%)	7 979 (26.00%)	
Usually	1 899 (6.01%)	80 (8.93%)	1 819 (5.93%)	
Getting up in morning				
Not very easy	10 573 (33.47%)	275 (30.69%)	10 298 (33.55%)	<.001
Fairly easy	13 409 (42.45%)	348 (38.84%)	13 061 (42.56%)	
Very easy	7 129 (22.57%)	261 (29.13%)	6 868 (22.38%)	
BMI				
<25	8 224 (26.04%)	197 (21.99%)	8 027 (26.15%)	.011
≥25	22 998 (72.81%)	675 (75.33%)	22 323 (72.73%)	
Smoking status				
Never/previous	25 375 (80.33%)	742 (82.81%)	24 633 (80.26%)	.019
Current	5 965 (18.88%)	141 (15.74%)	5 824 (18.98%)	
Alcohol drinker status				
Never	1 835 (5.81%)	75 (8.37%)	1 760 (5.73%)	<.001
Previous/current	29 539 (93.52%)	816 (91.07%)	28 723 (93.59%)	
Alcohol intake frequency				
Never	9 526 (30.16%)	317 (35.38%)	9 209 (30.01%)	<.001
<3 times/week	11 072 (35.05%)	260 (29.02%)	10 812 (35.23%)	
≥3 times/week	10 797 (34.18%)	316 (35.27%)	10 481 (34.15%)	
Waist circumference (mean±SD), cm	93.20 ± 14.75	96.12 ± 14.59	93.12 ± 14.75	<.001
Usual walking pace				
Slow pace	6 220 (19.69%)	276 (30.80%)	5 944 (19.37%)	<.001
Normal pace	16 043 (50.79%)	413 (46.09%)	15 630 (50.93%)	
Brisk pace	8 602 (27.23%)	171 (19.08%)	8 431 (27.47%)	
Stair climbs in 4 weeks				
≤5 times/day	11 436 (36.20%)	383 (42.75%)	11 053 (36.01%)	<.001
6–15 times/day	14 988 (47.45%)	368 (41.07%)	14 620 (47.64%)	
≥16 times/day	4 074 (12.90%)	93 (10.38%)	3 981 (12.97%)	
Phone use length				
≤1 year	5 576 (17.65%)	282 (31.47%)	5 294 (17.25%)	<.001
≥2 years	25 299 (80.09%)	575 (64.17%)	24 724 (80.56%)	
Playing computer games				
No	24 427 (77.33%)	742 (82.81%)	23 685 (77.17%)	<.001
Yes	6 998 (22.15%)	147 (16.41%)	6 851 (22.32%)	
Use of ultraviolet protection				
Always/don’t go out in sunshine	7 081 (22.42%)	181 (20.20%)	6 900 (22.48%)	<.001
Most of the time	9 909 (31.37%)	234 (26.12%)	9 675 (31.52%)	
Never/sometimes	14 112 (44.68%)	462 (51.56%)	13 650 (44.48%)	
Number in household (mean±SD), unit	2.24 ± 1.43	1.92 ± 1.08	2.25 ± 1.44	<.001
Able to confide				
≥1 time/week	20 555 (65.07%)	528 (58.93%)	20 027 (65.25%)	<.001
<1 time/week	9 938 (31.46%)	327 (36.50%)	9 611 (31.32%)	
Mood swings				
No	7 189 (22.76%)	241 (26.90%)	6 948 (22.64%)	.002
Yes	23 687 (74.99%)	628 (70.09%)	23 059 (75.13%)	
Long worry after shame				
No	11 523 (36.48%)	384 (42.86%)	11 139 (36.29%)	<.001
Yes	18 734 (59.31%)	452 (50.45%)	18 282 (59.57%)	
Duration of depression, mean (mean±SD), year	9.12 ± 5.78	5.36 ± 4.91	9.23 ± 5.76	<.001
Guilty feelings				
No	15 472 (48.98%)	535 (59.71%)	14 937 (48.67%)	<.001
Yes	14 950 (47.33%)	320 (35.71%)	14 630 (47.67%)	
Lethargy frequency in 2 weeks				
Not at all	7 107 (22.50%)	253 (28.24%)	6 854 (22.33%)	<.001
Several days	13 837 (43.81%)	354 (39.51%)	13 483 (43.93%)	
More than half the days	3 482 (11.02%)	84 (9.38%)	3 398 (11.07%)	
Nearly every day	6 087 (19.27%)	163 (18.19%)	5 924 (19.30%)	
Doctor visit for mental health				
No	5 461 (17.29%)	244 (27.23%)	5 217 (17.00%)	<.001
Yes	25 769 (81.58%)	634 (70.76%)	25 135 (81.90%)	
Pace-maker				
No	31 258 (98.96%)	874 (97.54%)	30 384 (99.00%)	.001
Yes	155 (0.49%)	11 (1.23%)	144 (0.47%)	

Continuous variables are presented as mean ± SD and categorical variables as number (%). *P* values were calculated based on Chi-square test for categorical variables and Mann-Whitney-Wilcoxon test for continuous variables

*Abbreviations*: *BMI* Body mass index, *SD *Standard deviation

### Development and validation of 27 variable-model

**Fig. 2:**
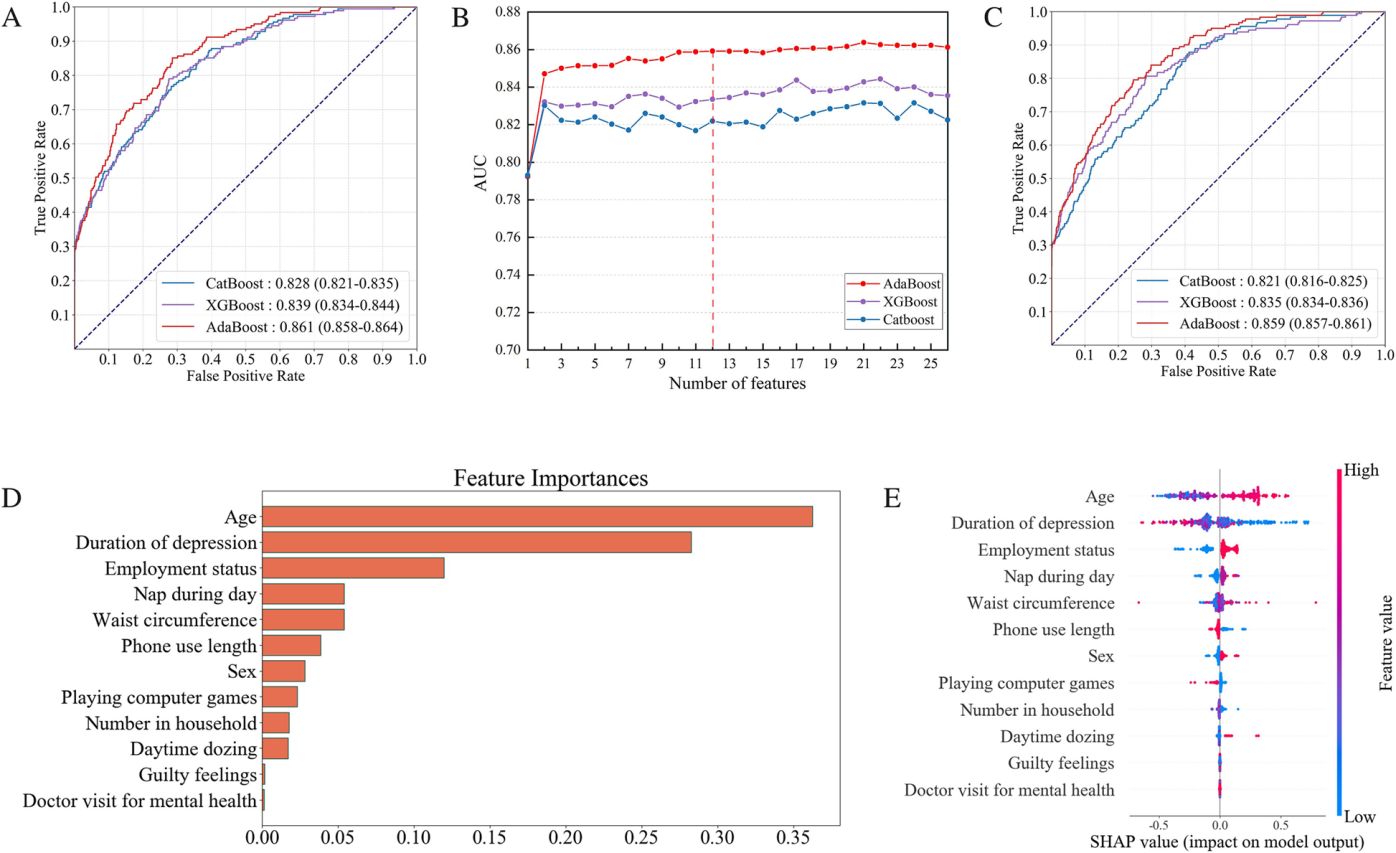
Model performance, feature selection results, and model interpretation. Area under the receiver operating characteristic curve (AUC) plots of different classifiers of 27 variables in Study 4. **A** The line chart depicts the change in AUC during the selection of a subset of features. 12 variables were finally remained for model optimization. **B** Area under the receiver operating characteristic curve (AUC) plots of different classifiers of 12 variables in Study 4. **C** The bar chart represents the sorted 12 variables based on their importance to the AdaBoost in Study 4. **D** SHAP visualization plot of the selected 12-variable simplified model. The specific effect of each variable on the model can be interpreted by its value magnitude (encoded by the color gradient) and tendency direction (on the horizontal axis) for the SHAP plot, where each datapoint represents an individual case's feature contribution in each row, with superimposed violin plots illustrating population-level effect size distributions (a red point represents a large feature value, and a blue point represents a small feature value). The horizontal displacement indicates effect direction (rightward: risk elevation; leftward: protective effect). When the SHAP value is greater than zero (right side), a larger value magnitude represents a higher risk of developing dementia. In contrast, when the SHAP value is less than zero (left side), a smaller value magnitude represents a larger likelihood against developing dementia. Taking age as an example, older participants (colored in red) were more likely to develop dementia (right side), and younger participants (colored in blue) had a larger likelihood against developing dementia (left side)

After the rigorous multi-stage selection procedure from the initial 190 candidate variables, ultimately retaining 27 variables that are easily available. These variables encompass established those that were validated to be associated with dementia. CatBoost, AdaBoost, and XGBoost were applied to evaluate the performance across eight distinct data partitioning schemes. As shown in Fig. 2A, comparative analysis revealed that AdaBoost achieved the optimal performance in Study 4 (AUC 0**.**861 ± 0**.**003), followed by XGBoost model (AUC 0**.**839 ± 0**.**005) and CatBoost model (AUC 0**.**828 ± 0**.**007).

Detailed performance metrics for Study 4 (Tables 2, 3) showed: 1) AdaBoost: mean accuracy of 98%, sensitivity of 100%, specificity of 98%, precision of 100%, and F1-score of 45**.**2%; 2) XGBoost: mean accuracy of 97**.**9%, sensitivity of 94**.**1%, specificity of 98%, precision of 94**.**1%, and F1-score of 44**.**7%; 3) CatBoost: mean accuracy of 97**.**9%, sensitivity of 96**.**6%, specificity of 97**.**9%, precision of 96**.**6%, and F1-score of 43**.**8%. All models achieved comparable performances, with testing set AUCs between 0**.**81 and 0**.**87 and training set AUCs between 0**.**80 and 0**.**85 (Supplementary eTable 4).

**Table 2:** Performance of AdaBoost model

	Accuracy	Sensitivity	Specificity	Precision	FI-score	AUC
27 Variables						
Study 1	0.979 ± 0.003	0.996 ± 0.013	0.979 ± 0.003	0.996 ± 0.013	0.397 ± 0.046	0.848 ± 0.027
Study 2	0.979 ± 0.001	0.991 ± 0.011	0.979 ± 0.001	0.991 ± 0.011	0.398 ± 0.028	0.849 ± 0.016
Study 3	0.978 (0.963–0.993)	1.000 (1.000-1.000)	0.978 (0.963–0.993)	1.000 (1.000-1.000)	0.365 (0.328–0.403)	0.851 (0.816–0.887)
Study 4	0.980 ± 0.000	1.000 ± 0.000	0.980 ± 0.000	1.000 ± 0.000	0.452 ± 0.003	0.861 ± 0.003
Study 5	0.977 ± 0.004	0.810 ± 0.102	0.979 ± 0.004	0.810 ± 0.102	0.396 ± 0.081	0.846 ± 0.030
Study 6	0.978 (0.966–0.991)	0.985 (0.974–0.995)	0.978 (0.966–0.991)	0.985 (0.974–0.995)	0.383 (0.352–0.414)	0.852 (0.823–0.881)
Study 7	0.979 ± 0.000	1.000 ± 0.000	0.979 ± 0.000	1.000 ± 0.000	0.449 ± 0.003	0.858 ± 0.004
Study 8	0.978 ± 0.004	0.862 ± 0.109	0.979 ± 0.004	0.862 ± 0.109	0.412 ± 0.056	0.841 ± 0.038
12 Variables						
Study 1	0.979 ± 0.003	0.996 ± 0.013	0.979 ± 0.003	0.996 ± 0.013	0.397 ± 0.046	0.848 ± 0.025
Study 2	0.979 ± 0.001	0.996 ± 0.009	0.979 ± 0.001	0.996 ± 0.009	0.398 ± 0.028	0.846 ± 0.016
Study 3	0.978 (0.963–0.993)	0.976 (0.960–0.992)	0.978 (0.963–0.993)	0.976 (0.960–0.992)	0.364 (0.327–0.401)	0.853 (0.817–0.888)
Study 4	0.980 ± 0.000	1.000 ± 0.000	0.980 ± 0.000	1.000 ± 0.000	0.452 ± 0.003	0.859 ± 0.002
Study 5	0.979 ± 0.003	0.906 ± 0.057	0.979 ± 0.003	0.906 ± 0.057	0.420 ± 0.082	0.833 ± 0.037
Study 6	0.978 (0.966–0.990)	0.970 (0.955–0.984)	0.978 (0.966–0.991)	0.970 (0.955–0.984)	0.382 (0.351–0.413)	0.849 (0.820–0.878)
Study 7	0.979 ± 0.000	0.995 ± 0.009	0.979 ± 0.000	0.995 ± 0.009	0.449 ± 0.003	0.855 ± 0.003
Study 8	0.979 ± 0.004	0.958 ± 0.052	0.979 ± 0.004	0.958 ± 0.052	0.419 ± 0.055	0.842 ± 0.028

*Abbreviations*: *AdaBoost* Adaptive Boosting

**Table 3:** Performance of XGBoost and CatBoost with 27 variables

	Accuracy	Sensitivity	Specificity	Precision	FI-score	AUC
Study1						
XGBoost	0.978 ± 0.003	0.931 ± 0.028	0.979 ± 0.003	0.931 ± 0.028	0.399 ± 0.043	0.827 ± 0.024
CatBoost	0.978 ± 0.003	0.934 ± 0.055	0.978 ± 0.003	0.934 ± 0.055	0.386 ± 0.044	0.818 ± 0.018
Study2						
XGBoost	0.978 ± 0.001	0.903 ± 0.052	0.979 ± 0.001	0.903 ± 0.052	0.394 ± 0.027	0.823 ± 0.013
CatBoost	0.978 ± 0.001	0.929 ± 0.020	0.978 ± 0.001	0.929 ± 0.020	0.385 ± 0.024	0.814 ± 0.013
Study3						
XGBoost	0.978 (0.962–0.993)	0.930 (0.904–0.956)	0.978 (0.963–0.993)	0.930 (0.904–0.956)	0.360 (0.323–0.397)	0.811 (0.773–0.850)
CatBoost	0.978 (0.962–0.993)	0.930 (0.904–0.956)	0.978 (0.963–0.993)	0.930 (0.904–0.956)	0.360 (0.323–0.397)	0.814 (0.776–0.853)
Study4						
XGBoost	0.979 ± 0.000	0.941 ± 0.029	0.980 ± 0.000	0.941 ± 0.029	0.447 ± 0.005	0.839 ± 0.005
CatBoost	0.979 ± 0.000	0.966 ± 0.013	0.979 ± 0.000	0.966 ± 0.013	0.438 ± 0.013	0.828 ± 0.007
Study5						
XGBoost	0.978 ± 0.004	0.835 ± 0.039	0.979 ± 0.003	0.835 ± 0.039	0.396 ± 0.089	0.838 ± 0.032
CatBoost	0.978 ± 0.004	0.952 ± 0.066	0.979 ± 0.004	0.952 ± 0.066	0.389 ± 0.095	0.843 ± 0.029
Study6						
XGBoost	0.978 (0.965–0.990)	0.880 (0.853–0.907)	0.978 (0.966–0.991)	0.880 (0.853–0.907)	0.384 (0.353–0.415)	0.824 (0.793–0.855)
CatBoost	0.978 (0.966–0.991)	0.985 (0.974–0.995)	0.978 (0.966–0.991)	0.985 (0.974–0.995)	0.383 (0.352–0.414)	0.814 (0.783–0.846)
Study7						
XGBoost	0.978 ± 0.000	0.923 ± 0.022	0.979 ± 0.000	0.923 ± 0.022	0.439 ± 0.006	0.832 ± 0.007
CatBoost	0.978 ± 0.000	0.914 ± 0.012	0.978 ± 0.000	0.914 ± 0.012	0.427 ± 0.007	0.830 ± 0.007
Study8						
XGBoost	0.978 ± 0.004	0.865 ± 0.166	0.979 ± 0.004	0.865 ± 0.166	0.407 ± 0.079	0.833 ± 0.027
CatBoost	0.978 ± 0.004	0.912 ± 0.064	0.979 ± 0.004	0.912 ± 0.064	0.402 ± 0.054	0.828 ± 0.025

*Abbreviations*: *XGBoost* eXtreme Gradient Boosting, *CatBoost* Categorical Boosting

### Development and validation of simplified model

To facilitate community generalization and clinical applicability, we develop a simplified model for web application through a forward feature subset selection algorithm based on Pearson’s correlation coefficient, ultimately retaining 12 variables. The 12 variables included age, sex, waist circumference, a doctor visit for mental health, guilty feelings, duration of depression, employment status, playing computer games, phone use length, number in household, daytime dozing, and nap during day.

As shown in Fig. 2B, model predictive capacity initially exhibited exponential enhancement with sequential incorporation of top-ranked variables. This progression transitioned to a stabilization phase characterized by performance plateauing accompanied by < 5% interquartile fluctuations upon integration of subsequent predictors. Ultimately, the algorithm converged at 12 optimally predictive features, with this parsimonious configuration achieving peak discriminative performance.

### Development and validation of simplified model

To facilitate community generalization and clinical applicability, we develop a simplified model for web application through a forward feature subset selection algorithm based on Pearson’s correlation coefficient, ultimately retaining 12 variables. The 12 variables included age, sex, waist circumference, a doctor visit for mental health, guilty feelings, duration of depression, employment status, playing computer games, phone use length, number in household, daytime dozing, and nap during day.

As shown in Fig. 2B, model predictive capacity initially exhibited exponential enhancement with sequential incorporation of top-ranked variables. This progression transitioned to a stabilization phase characterized by performance plateauing accompanied by < 5% interquartile fluctuations upon integration of subsequent predictors. Ultimately, the algorithm converged at 12 optimally predictive features, with this parsimonious configuration achieving peak discriminative performance.

As detailed in Fig. 2 C, the 12-variable simplified model maintained AdaBoost's diagnostic superiority in Study 4 (AUC 0**.**859 ± 0**.**002), followed by XGBoost model (AUC 0**.**835 ± 0**.**001) and CatBoost model (AUC 0**.**821 ± 0**.**005). Detailed performance metrics for Study 4 (Tables 2 and 4) showed: 1) AdaBoost: mean accuracy of 98%, sensitivity of 100%, specificity of 98%, precision of 100%, and F1-score of 45**.**2%; 2) XGBoost: mean accuracy of 97**.**9%, sensitivity of 91**.**6%, specificity of 98%, precision of 91**.**6%, and F1-score of 44**.**5%; 3) CatBoost: mean accuracy of 97**.**9%, sensitivity of 93%, specificity of 98%, precision of 93%, and F1-score of 44**.**4%. Additionally, all models have achieved decent performances with AUCs between 0**.**79 and 0.86 in the testing dataset and between 0**.**79 and 0**.**85 in the training dataset (Supplementary eTable 5). Cross-validation analyses confirmed the robustness of the simplified model, with testing set AUCs spanning 0.79–0.86 and training set AUCs ranging 0.79–0.85, indicating that the model retained generalizability (Supplementary Table 5).

**Table 4:** Performance of XGBoost and CatBoost with 12 variables

	Accuracy	Sensitivity	Specificity	Precision	FI-score	AUC
Study1						
XGBoost	0.978 ± 0.003	0.932 ± 0.061	0.979 ± 0.003	0.932 ± 0.061	0.396 ± 0.047	0.824 ± 0.025
CatBoost	0.978 ± 0.003	0.893 ± 0.061	0.978 ± 0.003	0.893 ± 0.061	0.381 ± 0.048	0.802 ± 0.016
Study2						
XGBoost	0.978 ± 0.001	0.945 ± 0.040	0.979 ± 0.001	0.945 ± 0.040	0.397 ± 0.025	0.822 ± 0.013
CatBoost	0.978 ± 0.001	0.908 ± 0.031	0.978 ± 0.001	0.908 ± 0.031	0.383 ± 0.021	0.801 ± 0.011
Study3						
XGBoost	0.978 (0.963–0.993)	0.952 (0.931–0.974)	0.978 (0.963–0.993)	0.952 (0.931–0.974)	0.362 (0.325–0.399)	0.820 (0.782–0.858)
CatBoost	0.978 (0.963–0.993)	0.952 (0.931–0.974)	0.978 (0.963–0.993)	0.952 (0.931–0.974)	0.362 (0.325–0.399)	0.795 (0.756–0.835)
Study4						
XGBoost	0.979 ± 0.000	0.916 ± 0.040	0.980 ± 0.000	0.916 ± 0.040	0.445 ± 0.006	0.835 ± 0.001
CatBoost	0.979 ± 0.000	0.930 ± 0.014	0.980 ± 0.000	0.930 ± 0.014	0.444 ± 0.004	0.821 ± 0.005
Study5						
XGBoost	0.976 ± 0.004	0.719 ± 0.059	0.979 ± 0.003	0.719 ± 0.059	0.395 ± 0.082	0.832 ± 0.024
CatBoost	0.978 ± 0.004	0.873 ± 0.097	0.979 ± 0.004	0.873 ± 0.097	0.383 ± 0.098	0.826 ± 0.014
Study6						
XGBoost	0.978 (0.966–0.990)	0.928 (0.906–0.949)	0.978 (0.966–0.991)	0.928 (0.906–0.949)	0.379 (0.348–0.410)	0.806 (0.774–0.837)
CatBoost	0.978 (0.966–0.990)	0.955 (0.938–0.973)	0.978 (0.966–0.991)	0.955 (0.938–0.973)	0.381 (0.350–0.412)	0.803 (0.771–0.835)
Study7						
XGBoost	0.978 ± 0.000	0.915 ± 0.027	0.979 ± 0.000	0.915 ± 0.027	0.439 ± 0.004	0.831 ± 0.005
CatBoost	0.978 ± 0.000	0.875 ± 0.031	0.979 ± 0.000	0.875 ± 0.031	0.429 ± 0.008	0.820 ± 0.004
Study8						
XGBoost	0.978 ± 0.005	0.850 ± 0.096	0.979 ± 0.004	0.850 ± 0.096	0.407 ± 0.055	0.819 ± 0.022
CatBoost	0.978 ± 0.004	0.848 ± 0.094	0.979 ± 0.004	0.848 ± 0.094	0.397 ± 0.062	0.815 ± 0.041

*Abbreviations*: *XGBoost* eXtreme Gradient Boosting, *CatBoost* Categorical Boosting

### Model interpretation and visualization

To elucidate the mechanistic contributions of the 12-variable panel to dementia risk prediction, we employed variable contribution ranking and SHapley Additive exPlanations (SHAP) plot analysis. Figure 2D shows the feature importance ranking of 12 variables in the optimized AdaBoost model. In our study, the contribution to the model in descending order were age, duration of depression, employment status, napping, waist circumference, phone use, sex, computer games, household number, daytime dozing, guilty feelings and doctor visit for mental health. Figure 2E demonstrates the SHAP value distribution through a beeswarm plot, quantitatively mapping each feature's non-linear impact on dementia probability. Analysis of SHAP value showed that age, duration of depression, and employment status had the greatest magnitude of effect, showing superior discrimination.

### Multivariable Cox regression model of 12 variables

Based on the 12 variables of the simplified model, we constructed a multivariate Cox proportional hazard regression analysis. In the final full variable model, unemployment status (HR, 1.51; 95% CI, 1.30–1.77), male gender (HR, 1.25; 95% CI, 1.09–1.43) and age (HR, 1.08; 95% CI, 1.07–1.09) were identified as risk factors for dementia (Supplementary eFigure 5). Conversely, three protective factors were identified: phone use length of two years or more (HR, 0.73; 95% CI, 0.63–0.85), having guilty feelings (HR, 0.78; 95% CI, 0.69–0.89) and doctor visit for mental health in the past (HR, 0.56; 95% CI, 0.48 to 0.65).. These findings were confirmed by SHAP value analysis, which showed consistency in identifying the directionality of protective/risk factors (Fig. 2E).

### Model comparison and robustness analysis

**Fig. 3:**
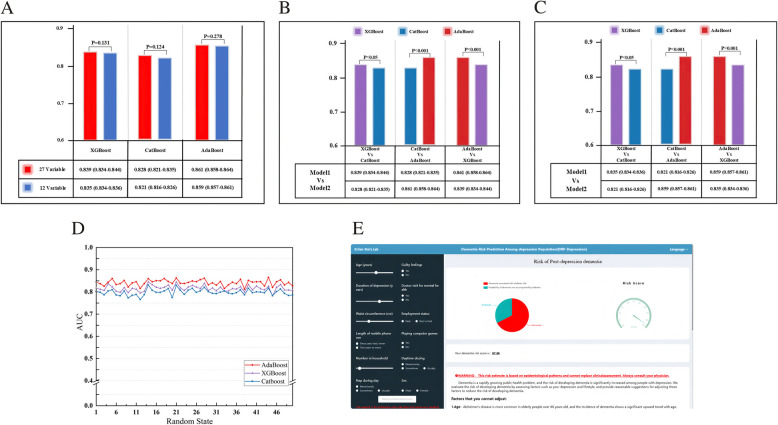
Model performance comparison charts, robustness analysis line charts, and Webpage interface of predictive model. **A** Comparison of models performance between 27 variables and 12 variables in Study 4 using the DeLong's test, and p < 0.05 suggested that there was a significant difference. **B** Comparison of the 3 classifiers (27 variables) in Study 4 using the DeLong's test, and p < 0.05 suggested that there was a significant difference. **C** Comparison of the 3 classifiers (12 variables) in Study 4 using the DeLong's test, and p < 0.05 suggested that there was a significant difference. **D** The line chart shows the robustness of the model after 50 random seed selections in Study 4. **E** The baseline characteristics can be entered on the left operation bar, and the right display bar shows the dementia risk in a pie chart and a dashboard form and provides reasonable prevention suggestions based on the baseline situation

In Study 4, comparative analyses of model performance by DeLong's test revealed no statistically significant differences in AUC values between the 12-variable and the 27-variable models (XGBoost, *p* = 0.131; CatBoost, *p* = 0.124; AdaBoost, *p* = 0.278; Fig. 3A; Supplementary eTable 6), demonstrating no significant superiority of the more complex 27-variable models (AUC range: 0.828–0.861) compared to the simplified 12-variable models (AUC range: 0.821–0.859), and the feasibility of simplified model through feature optimization.

DeLong's test was used to compare the AUC values between different machine learning classifiers for 12-variable (Fig. 3B) and 27-variable (Fig. 3C) models, respectively. Further comparative analysis revealed significant performance differences between different machine learning classifiers, with the AdaBoost demonstrating optimal performance in both the 12-variable an 0.27-variable models. Robustness analysis was used to evaluate the model stability of model performance through random subsampling calculations with 50 iterations. Figure 3D shows the AUC values within a narrow range of 0.8–0.86 across all classifiers. Therefore, we deployed the optimal simplified 12-variable model by the AdaBoost classifier to a web application for visualization and dementia risk assessment, named DRP-Depression (Fig. 3E).

### Webpage application deployment

The DRP-Depression model provides individualized dementia risk prediction for middle-aged and elderly individuals with depression. Baseline characteristic are inputted on the left-side operation bar. The upper right bar displays dementia risk via pie charts, while the middle-right panel displays detailed test reports. The lower-right interface generates reasonable personalized prevention recommendations that are derived based on the respective risk profiles provided by the tester. In addition, the DRP-Depression web interface includes prominent disclaimers clarifying that the tool is intended for risk stratification only, not clinical diagnosis.

Figure 3E illustrates a clinical case example of a 54-year-old male participant with a 19-year history of depression. The left panel displays his baseline characteristics, including a waist circumference of 105 cm, unemployment status, and single-person household composition. The DRP-Depression outputs calculated a dementia risk score of 67.36% for this individual, who subsequently received a clinical dementia diagnosis on April 15, 2017, during hospitalization.

The interface provides context-specific preventive guidance in the bottom-right section, such as ensure sufficient sleep and regular sleep patterns and adhering to prescribed depression treatment regimens. The web application is publicly accessible online at http://www.xijian-dai-lab.com.cn/modelIndex.

### Sensitivity analyses

Sensitivity analyses evaluated the temporal generalizability of the 12-variable simplified model by excluding cases with a recent diagnosis of depression (Supplementary eFigure 6): 

1. excluding cases diagnosed with depression within six-months: dementia group (*n*=787) vs dementia-free group (*n*=30691), AUC=0.838, (95%CI 0.832–0.845).
2. excluding cases diagnosed with depression within two-years: dementia group (*n*=630) vs dementia-free group (*n*=29534), AUC=0.802, (95%CI 0.797–0.807).

These findings demonstrated consistency with the primary analysis, reinforcing model robustness across temporal constraints.

## Discussion

In this prospective study of a large-scale UK Biobank cohort, several novel findings are noteworthy. First, we developed a dementia risk prediction model to identify high risk individuals among middle-aged and elderly adults with depression using machine learning algorithms applied to extensive UK Biobank data. The predictive model provides a straightforward and user-friendly interface for assessing dementia risk in depressed individuals. Second, our proposed dementia screening tool demonstrated a relatively high classification accuracy (98%) and an AUC value of 0.859 ± 0.002 in specific depressed population. Third, during the modeling process, feature importance ranking and SHAP value analysis were employed to evaluate the contribution and interpretability of each variable in the model output. Model stability was verified through eight distinct validation paradigms and robustness analyses. The Cox regression model and SHAP analysis showed consistent findings in identifying the directionality of protective/risk factors. Fourth, the variables included in the dementia screening tool are easily obtained, facilitating practical implementation and enhancing generalizability. Finally, we deployed the optimal-performing population-generalized model, named DRP-Depression, into an online publicly accessible web application.

To identify the optimal predictive model and systematically evaluate its stability and systematically, we employed rigorous dataset splitting strategies (e.g., transfer and validation) on large-scale data samples and implemented eight distinct validation paradigms incorporating three machine learning classifiers. Comprehensive evaluation showed that our machine learning framework has excellent stability (accuracy variance < 1%) and robust generalization capacity across heterogeneous datasets. The superior performance of the classifiers is mainly attributed to the ability of machine learning algorithms to process and analyze high-dimensional biomedical data through automated variable selection mechanism, particularly in capturing nonlinear associations and complex multi-variable interactions among variables [30]. Notably, these data-driven approaches effectively circumvent limitations inherent to conventional statistical approaches, which frequently impose restrictive parametric assumptions that compromise both predictive accuracy and model stability. Emerging evidence suggests that these computational methodologies are revolutionizing dementia diagnostics, with recent applications demonstrating high differential diagnostic accuracy in prodromal Alzheimer's disease cohorts when integrated with multimodal biomarkers [20, 31, 32].

Current diagnostic paradigms for dementia mainly rely on multimodal approaches including cognitive assessment scales (e.g., MMSE, MoCA), neuroimaging biomarkers (β-amyloid PET, FDG-PET), cerebrospinal fluid analysis (Aβ42/t-tau ratio), and emerging liquid biopsy techniques (peripheral blood autoantibody profiling, and neuron-derived exosome quantification). Notwithstanding these advances, early detection remains clinically challenging, as patients typically present for medical evaluation during advanced disease stages when irreversible neuropathological changes and significant comorbidities have already manifested. This diagnostic latency highlights the urgent need for validated predictive models to identify preclinical dementia risk in vulnerable populations [33]. Accumulating longitudinal evidences establish that depression may be a prodromal neuropsychiatric symptom and independent risk factor for dementia progression [12, 34]. Despite the identification of this etiologic link, there is still a conspicuous research gap in developing a mechanism-based screening framework, particularly for dementia conversion in depression cohorts. Current research on the progression of depression population to dementia mainly focuses on the epidemiological correlation of risk factors rather than the development of predictive models. For example, Xu et al., [35] found that adverse lifestyle factors were associated with a high risk of conversion from depression to dementia. Larsen et al., [36] found that men who developed depression before middle-aged and elderly had a higher risk of dementia in late-life.

Existing dementia risk stratification models for general populations exhibit substantial implementation barriers in real-world clinical settings. Kivipelto et al., developed a dementia risk score prediction model that took advantage of high age (> or = 47 years), low education (< 10 years), hypertension, hypercholesterolaemia, and obesity, and achieved a suboptimal predictive performance (AUC, 0.77; 95% CI 0.71–0.83). [18] Li et al. [37] used seven exposure factors to develop a dementia risk score model with a prediction accuracy of over 90%, but their model had relatively low discrimination (C statistic = 0.716). Our team developed a dementia risk score prediction model with better prediction performance [21]. The Rotterdam Study by Licher et al. [38] yielded a suboptimal predictive performance for predicting dementia (C-statistic, 0.78 [95% CI 0.75, 0.81]) using age, history of stroke, subjective memory decline, and need for assistance with finances or medication as predictors.. developed a machine learning-driven model for dementia risk stratification and deployed it into an online publicly accessible web application. Dementia risk prediction tools have three key translational advantages: 1) requires only 12 routinely documented and easily accessible clinical parameters, eliminating dependency on not directly available variables (e.g., cognitive assessment scales, neuroimaging biomarkers, CSF analysis, and emerging liquid biopsy techniques); 2) achieves superior predictive accuracy; 3) user-friendly interface, requiring short time for assessment.

Our longitudinal analysis revealed that chronological age emerged as the predominant risk determinant, which was consistent with established epidemiological patterns of exponential risk escalation beyond 65 years. Age remains the most significant established risk factor for dementia [39], with increasing age demonstrating a strong association with increased dementia risk progression [40]. Furthermore, emerging evidence suggests that aging process may be involved in the pathogenesis of dementia through dual mechanisms: dysbiosis of gut microbiota and progressive brain atrophy [41, 42]. Longitudinal analyses reveal that individuals diagnosed with depression at any life stage (early, middle, or late) exhibit a higher risk of dementia [6]. In this study cohort, we found that individuals with socioeconomic adversity (e.g., unemployed status) had a higher risk of dementia, which may be attributed to greater financial hardship, leading to more depression [43].

Our investigation revealed significant associations between modifiable risk factors and dementia susceptibility. Individuals who are prone to daytime sleepiness might have higher levels of longitudinal β-amyloid peptide deposition and thinner cortical thickness, which are considered as risk factors for dementia [44, 45], and this phenomenon may account for the higher risk of chronic sleepiness in persons with dementia. Our analysis further identified larger waist circumference as an independent predictor of dementia risk. Our analysis demonstrated that midlife overweight and obesity, when assessed using established anthropometric parameters for BMI and waist circumference, showed significant associations with dementia [46]. These findings confirm the durable predictive validity of traditional body composition measures for neurodegeneration risk stratification. Our findings also suggest that behavioral activities that relieve depressive mood could reduce the risk of dementia, such as receiving treatment from a psychiatrist, using a mobile phone, or playing computer games, can relieve psychological stress and reduce the risk of dementia. In addition, we found gender differences in the risk of dementia. Previous studies have shown that men have a higher risk of vascular dementia than women, which may be related to blood pressure [47] and cerebral perfusion levels [48] in men.

### Strengths and limitations

The major strengths of this study are the prospective design, large sample size, rigorous feature selection methods, easily accessible variables, clinical user-friendly interface, and superior predictive accuracy. However, our study has some limitations. First, the predominantly European ancestry composition of the cohort necessitate subsequent validation with a multiethnic cohort to confirm generalizability across different ethnicity. Second, some variables in the UK Biobank were assessed by human subjective evaluation, which may lead to misclassification. Third, all the study population were treated in hospital, which may cause population selection bias. Fourth, although we implemented very strict feature selection criteria, some potential variables may not have been taken into account. Fifth, because of the large difference in the number of people in the dementia, and non-dementia groups, there may be a class imbalance in the dataset.

## Conclusion

In this dementia diagnosis study in the middle-aged and elderly population with depression, we identified 27 clinically accessible predictors to develop and cross-validated an interpretable machine learning prediction framework to predict depression progression to dementia. We then developed and validated a simplified, reliable and clinically applicable machine learning-based prognostic tool and deployed it into an interoperable Web-based interface application to provide easy-to-promote in the community generalization. This cloud-based solution enables real-time dementia risk quantification in community and mental health settings, helps depression population identify their potential risk property, and provids personalized prevention recommendations through the intuitive interface. This pragmatic tool effectively bridges artificial intelligence innovation with clinical workflows, demonstrating significant potential for population-scale dementia prevention through its unique combination of algorithmic sophistication (XGBoost/SHAP integration) and practical implementability.

## Supplementary Information

Supplementary Material 1: eTable 1 The ICD-10 codes used for dementia and depression diagnosis in UKB. eTable 2 Candidate Variables Used in Machine Learning Models. eTable 3 Acquisition and evaluation of predictive factors. eTable 4 Model performance of training set in study 4, 5, 7, and 8 with 27 variables. eTable 5 Model performance of training set in study 4, 5, 7, and 8 with 12 variables. eTable 6 Use the DeLong's test to compare the AUC between the models with 27 variables and the models with 12 variables in Study4. eTable 7 The SHAP values of the 12 variables in the model were finally selected

Supplementary Material 2: eFigure 1 Correlation heatmap of included variables

Supplementary Material 3: eFigure 2 Flowchart of the selection of participants and variables. Note:"Variables are not easily available"refers to variables that cannot be obtained by asking or directly measuring

Supplementary Material 4: eFigure 3 27-variable model performance for the remaining seven data splitting designs. Area under the receiver operating characteristic curve (AUC) plots of different classifiers. Figure A-G depict Study 1, Study 2, Study 3, Study 5, Study 6, Study 7 and Study 8 respectively

Supplementary Material 5: eFigure 4 12-variable model performance for the remaining seven data splitting designs. Area under the receiver operating characteristic curve (AUC) plots of different classifiers. Figure A-G depict Study 1, Study 2, Study 3, Study 5, Study 6, Study 7 and Study 8 respectively

Supplementary Material 6: eFigure 5 Hazard ratios of included variables for dementia risk prediction model. The point estimate of the Cox proportional hazards model’s hazard ratio is depicted by the centre of the Forest plot, and the two-sided 95% confidence intervals are indicated by the error bars, the vertical line is the line of no effect

Supplementary Material 7: eFigure 6 AUC curve based on sensitivity analysis of the final selection model. A shows the AUC curve of the model after 6 months of excluding depression. B shows the AUC curve of the model after 2 years of excluding depression

Supplementary Material 8: eFigure 7 Enlarged version of the web interface diagram

## Data Availability

All the data used in this study are accessible on request from UK Biobank at: https://biobank.ndph.ox.ac.uk/ukb/. The codes used for data analysing in our study can be requested from the corresponding author (XJ. D).
