# Supplementary material for: Development and validation of a novel predictive model for dementia risk in middle-aged and elderly depression individuals: a large and longitudinal machine learning cohort study
Source: Alzheimers Res Ther. 2025 May 13;17:103. doi: 10.1186/s13195-025-01750-6 (PMC12070709; doi:10.1186/s13195-025-01750-6)
Supplement: Supplementary file 1 — Supplementary Material 1: eTable 1 The ICD-10 codes used for dementia and depression diagnosis in UKB. eTable 2 Candidate Variables Used in Machine Learning Models. eTable 3 Acquisition and evaluation of predictive factors. eTable 4 Model performance of training set in study 4, 5, 7, and 8 with 27 variables. eTable 5 Model performance of training set in study 4, 5, 7, and 8 with 12 variables. eTable 6 Use the DeLong's test to compare the AUC between the models with 27 variables and the models with 12 variables in Study4. eTable 7 The SHAP values of the 12 variables in the model were finally selected [file 13195_2025_1750_MOESM1_ESM.docx]

**Supplement-eTable1 to 7**

**eTable 1** **The ICD-10 codes used for dementia and depression diagnosis in UKB**

| **Diagnosis** | **Codes** |
| --- | --- |
| **Dementia** | F00.0, F00.1, F00.2, F00.9, F01.0, F01.1, F01.2, F01.3, F01.8, F01.9, F02.0, F02.1, F02.2, F02.3, F02.4, F02.8, F03, F04, F05.1, G30.0, G30.1, G30.8, G30.9 |
| **Depression** | F32.0, F32.1, F32.2, F32.3, F32.8, F32.9, F33.0, F33.1, F33.2, F33.3, F33.4, F33.8, F33.9, F41.2, T43.0 |

**eTable 2 Candidate Variables Used in Machine Learning Models**

| **Main category** | **Field ID** |
| --- | --- |
| **Demographic characteristics**  **(n =5)** | 31(Sex), 3526(Mother’s age at death), 1807(Father’s age at death), 21022(Age), 709(Number in household) |
| **lifestyle and environmental exposures**  **(n = 51)** | 874(Duration of walks), 924(Usual walking pace), 22032(IPAQ activity group), 22035(At or above moderate/vigorous recommendation), 22038(MET minutes per week for moderate activity), 22039(MET minutes per week for vigorous activity), 943(Stair climbs in 4 weeks), 6164(Types of physical activity in last 4 weeks), 1050(Time spend outdoors in summer), 1060(Time spent outdoors in winter), 1070(Time spent watching television (TV)), 1080(Time spent using computer), 1090(Time spent driving), 1737(Childhood sunburn occasions), 2267(Use of ultraviolet protection), 2277(Frequency of solarium/sunlamp use), 864(Number of days/week walked 10+ minutes), 884(Number of days/week of moderate physical activity 10+ minutes), 894(Duration of moderate activity), 904(Number of days/week of vigorous physical activity 10+ minutes), 914(Duration of vigorous activity), 1110(Phone use length), 20116(Smoking status), 1329(Oily fish intake), 1339(Non-oily fish intake), 1349(Processed meat intake), 1369(Beef intake), 1379(Lamb/mutton intake), 1389(Pork intake), 1438(Bread intake), 1448(Bread type), 1458(Cereal intake), 1468(Cereal type), 20117(Alcohol drinker status), 24007(Particulate matter air pollution (pm2.5) absorbance; 2010), 971(Frequency of walking for pleasure in last 4 weeks), 981(Duration walking for pleasure), 2237(Playing computer games), 1239(Current tobacco smoking), 1259(Smoking/smokers in household), 1548(Variation in diet), 3506(Smoking compared to 10 years previous), 3731(Former alcohol drinker), 22040(Summed MET minutes per week for all activity), 1289(Cooked vegetable intake), 1299(Salad / raw vegetable intake), 1309(Fresh fruit intake), 1319(Dried fruit intake), 1558(Alcohol intake frequency), 1618(Alcohol usually taken with meals), 2207(Wears glasses or contact lenses) |
| **Sleep phenotypes**  **(n =8)** | 1160(Sleep duration), 1180(Morning/evening person (chronotype)), 1190(Nap during day), 1200(Sleeplessness), 1210(Snoring), 1220(Daytime dozing), 1170(Getting up in morning), 826(Job involves shift work) |
| **Socioeconomic status**  **(n=10)** | 738(Average total household income before tax), 777(Frequency of travelling from home to job workplace), 796(Distance between home and job workplace), 6143(Transport type for commuting to job workplace), 767(Length of working week for main job), 816(Job involves heavy manual or physical work), 3426(Job involves night shift work), 6142(Employment status), 4825(Noisy workplace), 6138(Qualifications) |
| **Medications and medical history**  **(n = 53)** | 21065(Family history of IBS), 2443(Diabetes diagnosed by doctor), 2976(Age diabetes diagnosed), 5890(Which eye(s) affected by diabetes-related eye disease), 2010(Suffer from 'nerves'), 2178(Overall health rating), 2188(Long-standing illness, disability or infirmity), 2335(Chest pain or discomfort), 2415(Had major operations), 5408(Which eye(s) affected by amblyopia (lazy eye)), 5419(Which eye(s) affected by injury or trauma resulting in loss of vision), 6119(Which eye(s) affected by glaucoma), 2247(Hearing difficulty/problems), 2473(Other serious medical condition/disability diagnosed by doctor), 3393(Hearing aid user), 4041(Gestational diabetes only), 4792(Cochlear implant), 4803(Tinnitus), 5610(Which eye(s) affected by presbyopia), 6205(Which eye(s) affected by strabismus (squint)), 2453(Cancer diagnosed by doctor), 2844(Had other major operations), 3079(Pace-maker), 6014(Doctor restricts physical activity due to heart condition), 5441(Which eye(s) are affected by cataract), 5324(Ever had cataract surgery), 2227(Other eye problems), 2296(Falls in the last year), 5452(Leg pain when standing still or sitting), 5463(Leg pain in calf/calves), 5474(Leg pain when walking uphill or hurrying), 5485(Leg pain when walking normally), 5496(Leg pain when walking ever disappears while walking), 5934(Which eye(s) affected by other serious eye condition), 6149(Mouth/teeth dental problems), 6156(Manic/hyper symptoms), 84(Cancer year/age first occurred), 87(Non-cancer illness year/age first occurred), 92(Operation year/age first occurred), 6153(Medication for cholesterol, blood pressure, diabetes, or take exogenous hormones), 6177(Medication for cholesterol, blood pressure or diabetes), 5540(Surgery/amputation of toe or leg), 5663(Length of longest manic/irritable episode), 2492(Taking other prescription medications), 6015(Chest pain felt during physical activity), 6016(Chest pain felt outside physical activity), 5183(Current eye infection), 5325(Ever had refractive laser eye surgery), 5326(Ever had surgery for glaucoma or high eye pressure), 5327(Ever had laser treatment for glaucoma or high eye pressure), 5328(Ever had corneal graft surgery), 5529(Surgery on leg arteries (other than for varicose veins)) |
| **Laboratory tests**  **(n=23)** | 30000(White blood cell (leukocyte) count), 30120(Lymphocyte count), 30130(Monocyte count), 30140(Neutrophill count), 30630(Apolipoprotein A), 30640(Apolipoprotein B), 30690(Cholesterol), 30710(C-reactive protein), 30760(HDL cholesterol), 30780(LDL direct), 30790(Lipoprotein A), 30870(Triglycerides), 30740(Glucose), 30750(Glycated haemoglobin (HbA1c)), 30600(Albumin), 30610(Alkaline phosphatase), 30620(Alanine aminotransferase), 30650(Aspartate aminotransferase), 30770(IGF-1), 30020(Haemoglobin concentration), 30670(Urea), 30720(Cystatin C), 30730(Gamma glutamyltransferase) |
| **Physical measures**  **(n=10)** | 21001(BMI), 46(Hand grip strength (left)), 47(Hand grip strength (right)), 20016(Fluid intelligence score), 48(Waist circumference), 50(Standing height), 2306(Weight change compared with 1 year ago), 6017(Able to walk or cycle unaided for 10 minutes), 6019(ECG/bike method for fitness test), 6034(Target heart rate achieved) |
| **Social and psychological factors**  **(n=29)** | 20458(General happiness), 1031(Frequency of friend/family visits), 2110(Able to confide), 20127(Neuroticism score), 20435(Difficulty concentrating during worst depression), 20436(Fraction of day affected during worst episode of depression), 20437(Thoughts of death during worst depression), 20439(Frequency of depressed days during worst episode of depression), 20440(Impact on normal roles during worst period of depression), 20441(Ever had prolonged loss of interest in normal activities), 20446(Ever had prolonged feelings of sadness or depression), 20450(Feelings of worthlessness during worst period of depression), 20510(Recent feelings of depression), 20532(Did your sleep change?), 20536(Weight change during worst episode of depression), 1920(Mood swings), 2000(Long worry after shame), 2020(Loneliness, isolation), 2030(Guilty feelings), 2040(Risk taking), 2050(Frequency of depressed mood in last 2 weeks), 2060(Frequency of unenthusiasm / disinterest in last 2 weeks), 2070(Frequency of tenseness / restlessness in last 2 weeks), 2080(Lethargy frequency in 2 weeks), 2090(Doctor visit for mental health), 2100(Seen a psychiatrist for nerves, anxiety, tension or depression), 4501(Non-accidental death in close genetic family), 6145(Illness, injury, bereavement, stress in last 2 years), 6160(Leisure/social activities) |
| **Self-generated variable**  **(n=1)** | Duration of depression |
| **Date of attending assessment center** | 53(Date of attending assessment centre) |

**eTable 3 Acquisition and evaluation of predictive factors**

| **Variables** | **Ways to acquire the variables** | [**Possible answers and answer coding**](http://translate.baidu.com/) |
| --- | --- | --- |
| **Age** | The age of the participant on the day they attended an Initial Assessment Centre and self-reported by the participant. | A continuous number |
| **Sex** | Acquired from central registry at recruitment, but in some cases updated by the participant. | 0: Female;  1: Male |
| **Employment status** | The touchscreen question "Which of the following describes your current situation?" | 0: In paid;  1: Not in paid |
| **Sleep duration^1^** | The touchscreen question "About how many hours sleep do you get in every 24 hours? (please include naps)" | 0: 7-8 h;  1: ≤6 h;  2: ≥9 h |
| **Nap during day** | The touchscreen question "Do you have a nap during the day?" | 0: Never/rarely;  1: Sometimes;  2: Usually |
| **Sleeplessness** | The touchscreen question "Do you have trouble falling asleep at night or do you wake up in the middle of the night?" | 0: Never;  1: Sometimes/ usually |
| **Daytime dozing** | The touchscreen question "How likely are you to doze off or fall asleep during the daytime when you don't mean to? (e.g., when working, reading or driving)" | 0: Never;  1: Sometimes;  2: Usually |
| **Getting up in morning** | The touchscreen question "On an average day, how easy do you find getting up in the morning?" | 0: Not very easy;  1: Fairly easy;  2: Very easy |
| **BMI** | BMI was calculated by dividing weight in kilograms by height in meters squared during the initial visit to the assessment centers. BMI value here is constructed from height and weight measured during the initial Assessment Centre visit. | 0: <25;  1: ≥25 |
| **Smoking status** | This field summarizes the current/past smoking status of the participant. | 0: Never/previous;  1: Current |
| **Alcohol drinker status** | This field summarizes the current/past alcohol drinker status of the participant. | 0: Never;  1: Previous/current |
| **Alcohol intake frequency** | The touchscreen question "About how often do you drink alcohol?" | 0: Never;  1: <3 times/week;  2: ≥3 times/week |
| **Waist circumference** | Self-reported by participants. | A continuous number |
| **Usual walking pace** | The touchscreen question "How would you describe your usual walking pace?" | 0: Slow pace;  1: Normal pace;  2: Brisk pace |
| **Stair climbs in 4 weeks** | The touchscreen question "At home, during the last 4 weeks, about how many times a DAY do you climb a flight of stairs? (approx. 10 steps)" | 0: ≤5 times/day;  1: 6-15 times/day;  2: ≥16 times/day |
| **Phone use length** | The touchscreen question "For approximately how many years have you been using a mobile phone at least once per week to make or receive calls?" | 0: ≤1 year;  1: ≥2 years |
| **Playing computer games** | The touchscreen question "Do you play computer games?" | 0: No;  1: Yes |
| **Use of ultraviolet protection** | The touchscreen question "Do you wear sun protection (e.g., sunscreen lotion, hat) when you spend time outdoors in the summer?" | 0: Always/don’t go out in sunshine;  1: Most of the time;  2: Never/sometimes |
| **Number in household** | The touchscreen question "Including yourself, how many people are living together in your household? (Include those who usually live in the house such as students living away from home during term, partners in the armed forces or professions such as pilots)" | A continuous number |
| **Able to confide** | The touchscreen question "How often are you able to confide in someone close to you?" | 0: ≥1 time/week;  1: <1 time/week |
| **Mood swings** | The touchscreen question "Does your mood often go up and down?" | 0: No;  1: Yes |
| **Long worry after shame** | The touchscreen question "Do you worry too long after an embarrassing experience?" | 0: No;  1: Yes |
| **Duration of depression^2^** | Self-generated variable. | A continuous number |
| **Guilty feelings** | The touchscreen question "Are you often troubled by feelings of guilt?" | 0: No;  1: Yes |
| **Lethargy frequency in 2 weeks** | The touchscreen question "Over the past two weeks, how often have you felt tired or had little energy?" | 0: Not at all;  1: Several days;  2: More than half the days;  3: Nearly every day |
| **Doctor visit for mental health** | The touchscreen question "Have you ever seen a general practitioner for nerves, anxiety, tension or depression?" | 0: No;  1: Yes |
| **Pace-maker** | Participants asked by interviewer if they have a pace-maker before the body impedance measures. | 0: No;  1: Yes |

**Notes:1. The sleep duration is a continuous variable that we manually categorize into three groups.**

**2. The duration of depression is calculated by clinical doctors for each subject, starting from the time of diagnosis of depression until the time of diagnosis of dementia (for the dementia-free group, until the end of the follow-up period).**

**Abbreviations: BMI, Body mass index.**

**eTable 4 Model performance of training set in study 4, 5, 7, and 8** **with 27 variables**

|  | **Accuracy** | **Sensitivity** | **Specificity** | **Precision** | **FI-score** | **AUC** |
| --- | --- | --- | --- | --- | --- | --- |
| **Study4** |  |  |  |  |  |  |
| AdaBoost | 0.978 ± 0.001 | 0.994 ± 0.011 | 0.978 ± 0.002 | 0.994 ± 0.011 | 0.383 ± 0.050 | 0.843 ± 0.011 |
| XGBoost | 0.978 ± 0.001 | 0.915 ± 0.033 | 0.978 ± 0.002 | 0.915 ± 0.033 | 0.382 ± 0.046 | 0.816 ± 0.015 |
| CatBoost | 0.977 ± 0.001 | 0.911 ± 0.061 | 0.978 ± 0.002 | 0.911 ± 0.061 | 0.356 ± 0.046 | 0.810 ± 0.012 |
| **Study5** |  |  |  |  |  |  |
| AdaBoost | 0.978 ± 0.001 | 0.985 ± 0.029 | 0.978 ± 0.001 | 0.985 ± 0.029 | 0.390 ± 0.034 | 0.843 ± 0.014 |
| XGBoost | 0.978 ± 0.001 | 0.883 ± 0.033 | 0.979 ± 0.001 | 0.883 ± 0.033 | 0.385 ± 0.029 | 0.806 ± 0.006 |
| CatBoost | 0.978 ± 0.001 | 0.913 ± 0.043 | 0.978 ± 0.001 | 0.913 ± 0.043 | 0.377 ± 0.031 | 0.803 ± 0.005 |
| **Study7** |  |  |  |  |  |  |
| AdaBoost | 0.979 ± 0.001 | 0.983 ± 0.021 | 0.979 ± 0.001 | 0.983 ± 0.021 | 0.370 ± 0.049 | 0.830 ± 0.015 |
| XGBoost | 0.978 ± 0.001 | 0.872 ± 0.032 | 0.978 ± 0.001 | 0.872 ± 0.032 | 0.362 ± 0.046 | 0.813 ± 0.013 |
| CatBoost | 0.978 ± 0.001 | 0.898 ± 0.047 | 0.978 ± 0.001 | 0.898 ± 0.047 | 0.343 ± 0.043 | 0.804 ± 0.019 |
| **Study8** |  |  |  |  |  |  |
| AdaBoost | 0.978 ± 0.003 | 0.970 ± 0.047 | 0.978 ± 0.002 | 0.970 ± 0.047 | 0.384 ± 0.068 | 0.836 ± 0.022 |
| XGBoost | 0.978 ± 0.002 | 0.875 ± 0.041 | 0.978 ± 0.002 | 0.875 ± 0.041 | 0.380 ± 0.063 | 0.808 ± 0.016 |
| CatBoost | 0.978 ± 0.002 | 0.927 ± 0.047 | 0.978 ± 0.002 | 0.927 ± 0.047 | 0.365 ± 0.050 | 0.801 ± 0.024 |

**Abbreviations: AdaBoost, Adaptive Boosting; XGBoost, eXtreme Gradient Boosting; CatBoost,** **Categorical Boosting.**

**eTable5 Model performance of training set in study 4, 5, 7, and 8** **with 12 variables**

|  | **Accuracy** | **Sensitivity** | **Specificity** | **Precision** | **FI-score** | **AUC** |
| --- | --- | --- | --- | --- | --- | --- |
| **Study4** |  |  |  |  |  |  |
| AdaBoost | 0.978 ± 0.002 | 0.992 ± 0.015 | 0.978 ± 0.002 | 0.992 ± 0.015 | 0.381 ± 0.049 | 0.842 ± 0.012 |
| XGBoost | 0.978 ± 0.002 | 0.876 ± 0.072 | 0.978 ± 0.002 | 0.876 ± 0.072 | 0.376 ± 0.053 | 0.814 ± 0.009 |
| CatBoost | 0.977 ± 0.001 | 0.878 ± 0.075 | 0.978 ± 0.002 | 0.878 ± 0.075 | 0.347 ± 0.047 | 0.797 ± 0.011 |
| **Study5** |  |  |  |  |  |  |
| AdaBoost | 0.978 ± 0.001 | 0.990 ± 0.020 | 0.978 ± 0.001 | 0.990 ± 0.020 | 0.390 ± 0.034 | 0.841 ± 0.017 |
| XGBoost | 0.978 ± 0.001 | 0.922 ± 0.030 | 0.978 ± 0.001 | 0.922 ± 0.030 | 0.387 ± 0.031 | 0.809 ± 0.012 |
| CatBoost | 0.978 ± 0.001 | 0.885 ± 0.048 | 0.978 ± 0.001 | 0.885 ± 0.048 | 0.375 ± 0.032 | 0.786 ± 0.006 |
| **Study7** |  |  |  |  |  |  |
| AdaBoost | 0.979 ± 0.001 | 1.000 ± 0.000 | 0.978 ± 0.001 | 1.000 ± 0.000 | 0.369 ± 0.046 | 0.830 ± 0.012 |
| XGBoost | 0.978 ± 0.001 | 0.889 ± 0.050 | 0.978 ± 0.001 | 0.889 ± 0.050 | 0.361 ± 0.045 | 0.815 ± 0.011 |
| CatBoost | 0.977 ± 0.001 | 0.858 ± 0.037 | 0.978 ± 0.001 | 0.858 ± 0.037 | 0.336 ± 0.043 | 0.796 ± 0.010 |
| **Study8** |  |  |  |  |  |  |
| AdaBoost | 0.978 ± 0.003 | 0.975 ± 0.050 | 0.978 ± 0.002 | 0.975 ± 0.050 | 0.383 ± 0.068 | 0.832 ± 0.021 |
| XGBoost | 0.978 ± 0.002 | 0.878 ± 0.066 | 0.978 ± 0.002 | 0.878 ± 0.066 | 0.380 ± 0.069 | 0.799 ± 0.024 |
| CatBoost | 0.978 ± 0.002 | 0.917 ± 0.044 | 0.978 ± 0.002 | 0.917 ± 0.044 | 0.370 ± 0.061 | 0.790 ± 0.019 |

**Abbreviations: AdaBoost, Adaptive Boosting; XGBoost, eXtreme Gradient Boosting; CatBoost,** **Categorical Boosting.**

**eTable 6 Use the DeLong's test to compare the AUC between the models with 27 variables and the models with 12 variables in Study4**

|  | **27-variable models’ AUCs** | **12-variable models’ AUCs** | ***p*-value** |
| --- | --- | --- | --- |
| **AdaBoost** | 0.861 ± 0.003 | 0.859 ± 0.002 | .278 |
| **XGBoost** | 0.839 ± 0.005 | 0.835 ± 0.001 | .131 |
| **CatBoost** | 0.828 ± 0.007 | 0.821 ± 0.005 | .124 |

**Abbreviations: AdaBoost, Adaptive Boosting; XGBoost, eXtreme Gradient Boosting; CatBoost,** **Categorical Boosting.**

**eTable 7 The SHAP values of the 12 variables in the model were finally selected**

| **Variables** | **SHAP value** | **Variables** | **SHAP value** |
| --- | --- | --- | --- |
| **Age** | 0.362 | **Sex** | 0.028 |
| **Duration of depression** | 0.282 | **Plays computer games** | 0.023 |
| **Employment status** | 0.120 | **Number in household** | 0.018 |
| **Nap during day** | 0.054 | **Daytime dozing** | 0.017 |
| **Waist circumference** | 0.054 | **Guilty feelings** | 0.002 |
| **Phone use length** | 0.039 | **Doctor visit for mental health** | 0.001 |
