# Supplementary figures and images for: Development and validation of a novel predictive model for dementia risk in middle-aged and elderly depression individuals: a large and longitudinal machine learning cohort study
Source: Alzheimers Res Ther. 2025 May 13;17:103. doi: 10.1186/s13195-025-01750-6 (PMC12070709; doi:10.1186/s13195-025-01750-6)

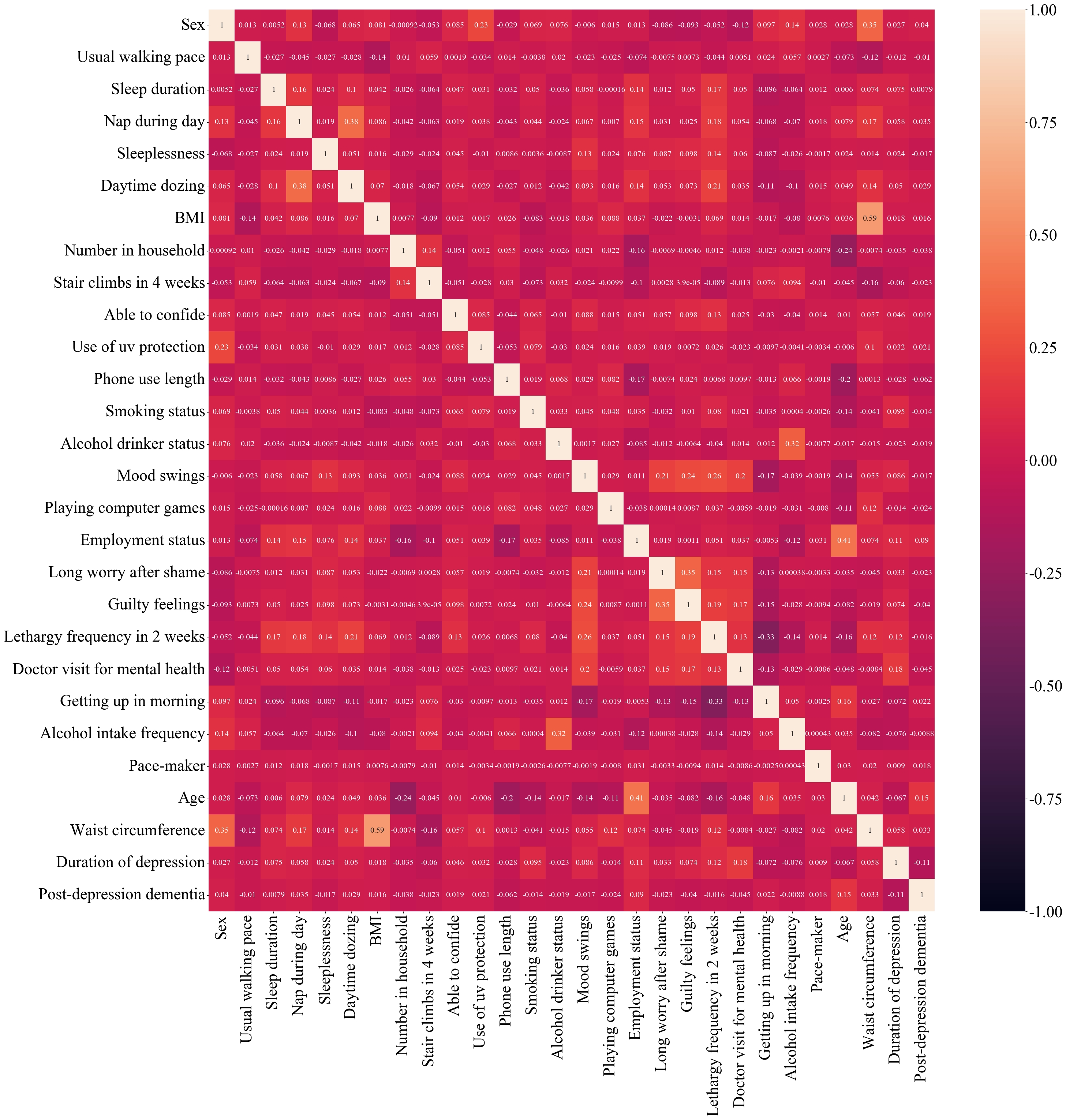

Supplement: Supplementary file 2 — Supplementary Material 2: eFigure 1 Correlation heatmap of included variables [file 13195_2025_1750_MOESM2_ESM.jpg]

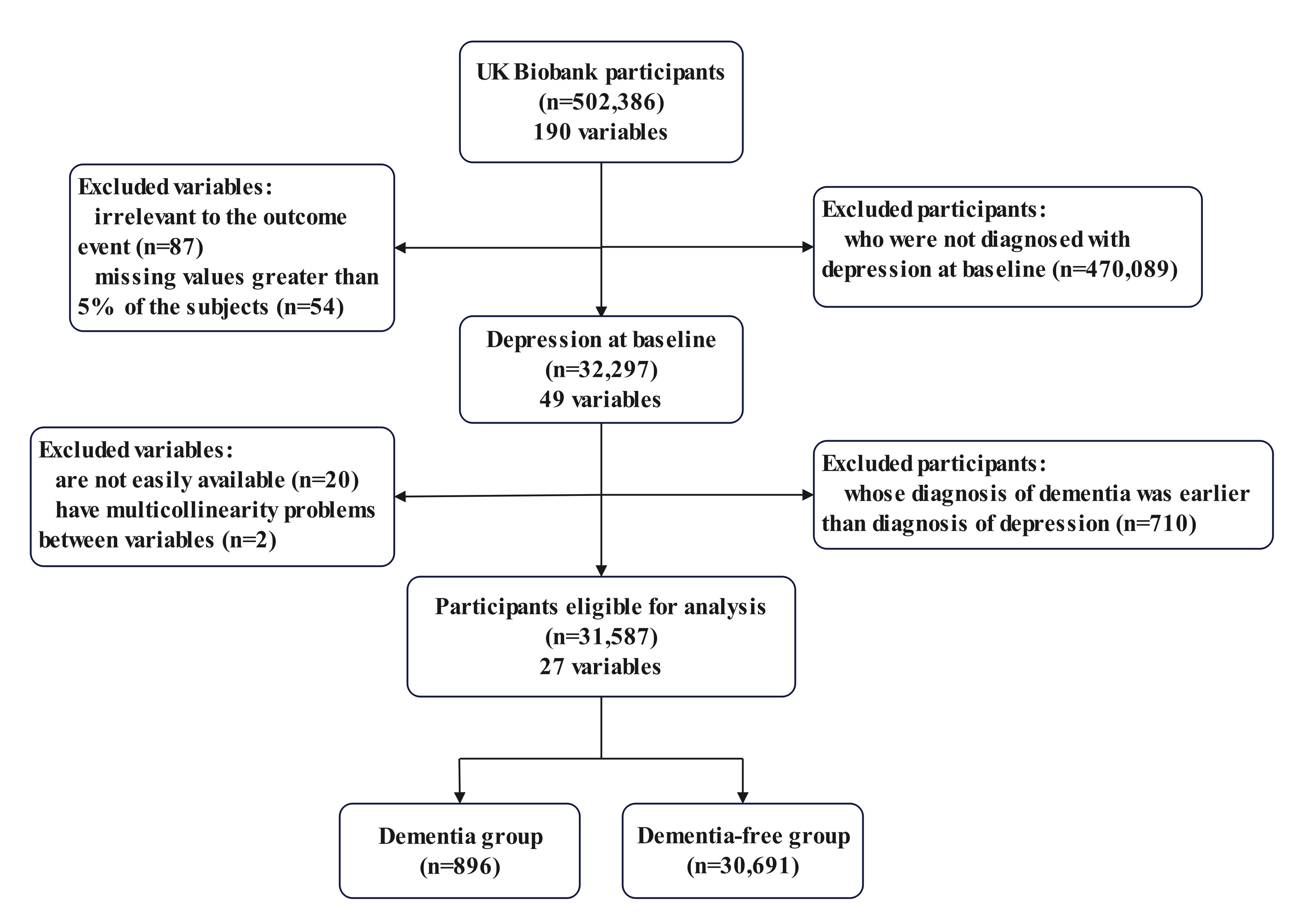

Supplement: Supplementary file 3 — Supplementary Material 3: eFigure 2 Flowchart of the selection of participants and variables. Note:"Variables are not easily available"refers to variables that cannot be obtained by asking or directly measuring [file 13195_2025_1750_MOESM3_ESM.jpg]

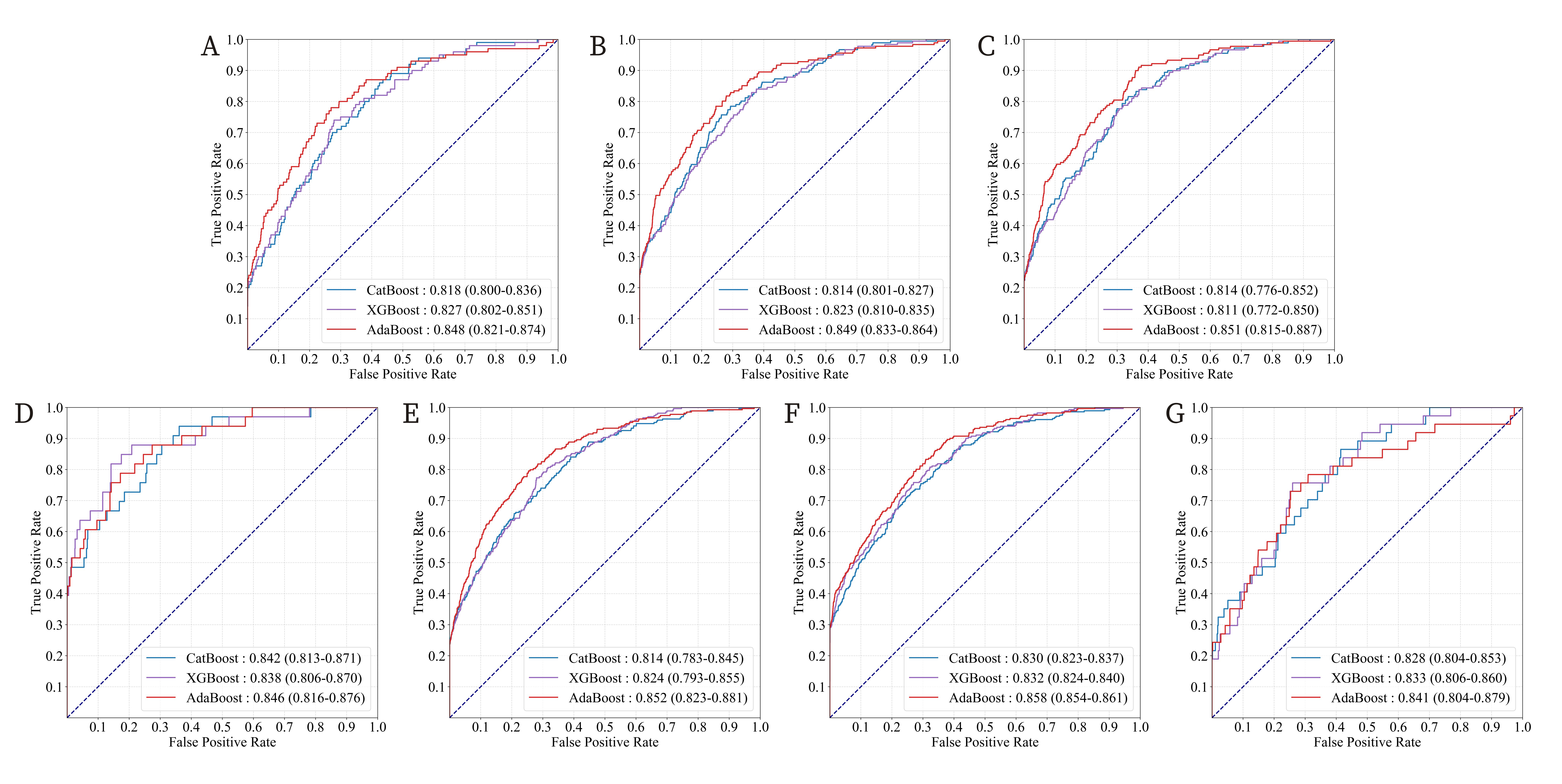

Supplement: Supplementary file 4 — Supplementary Material 4: eFigure 3 27-variable model performance for the remaining seven data splitting designs. Area under the receiver operating characteristic curve (AUC) plots of different classifiers. Figure A-G depict Study 1, Study 2, Study 3, Study 5, Study 6, Study 7 and Study 8 respectively [file 13195_2025_1750_MOESM4_ESM.jpg]

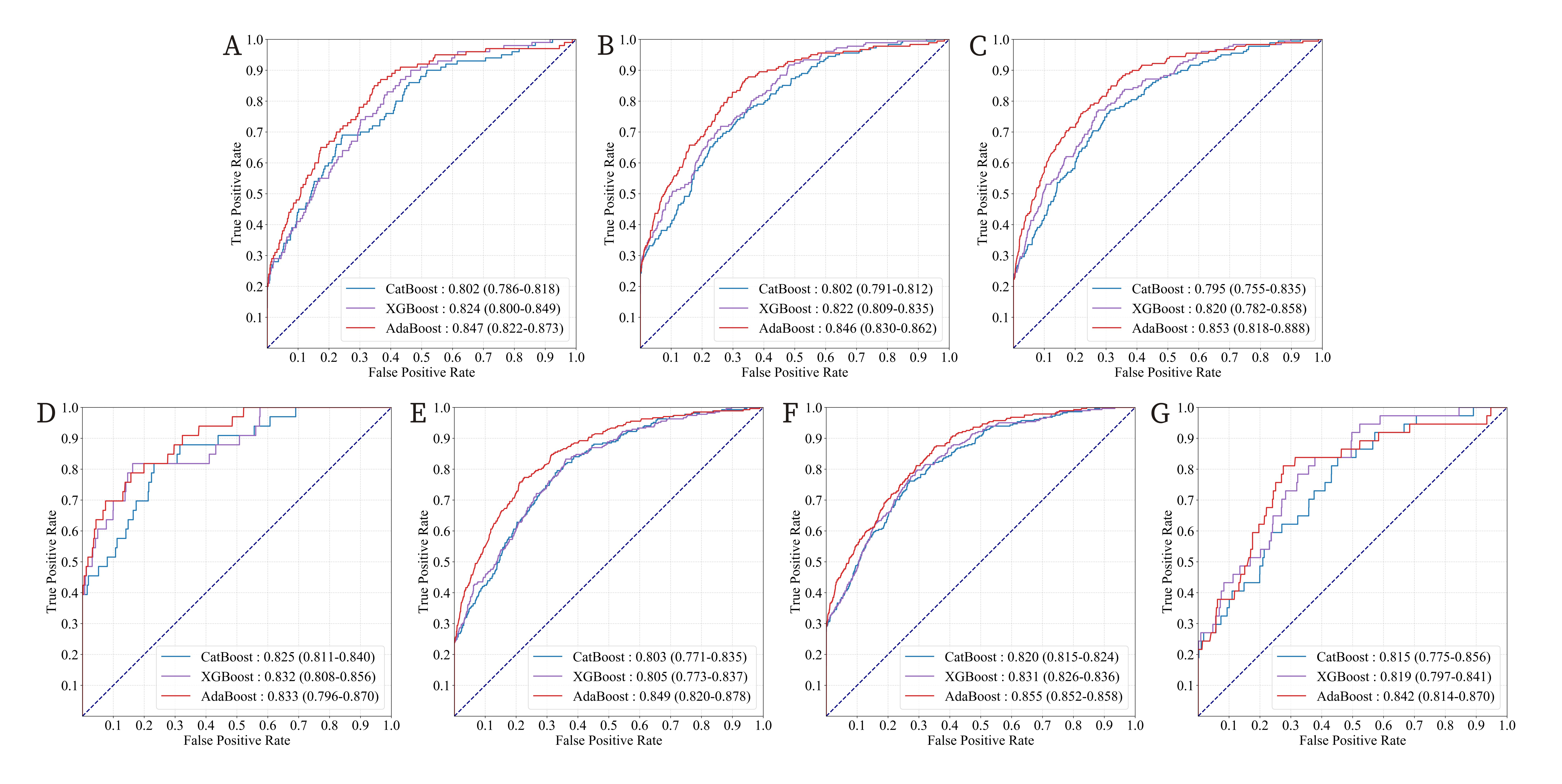

Supplement: Supplementary file 5 — Supplementary Material 5: eFigure 4 12-variable model performance for the remaining seven data splitting designs. Area under the receiver operating characteristic curve (AUC) plots of different classifiers. Figure A-G depict Study 1, Study 2, Study 3, Study 5, Study 6, Study 7 and Study 8 respectively [file 13195_2025_1750_MOESM5_ESM.jpg]

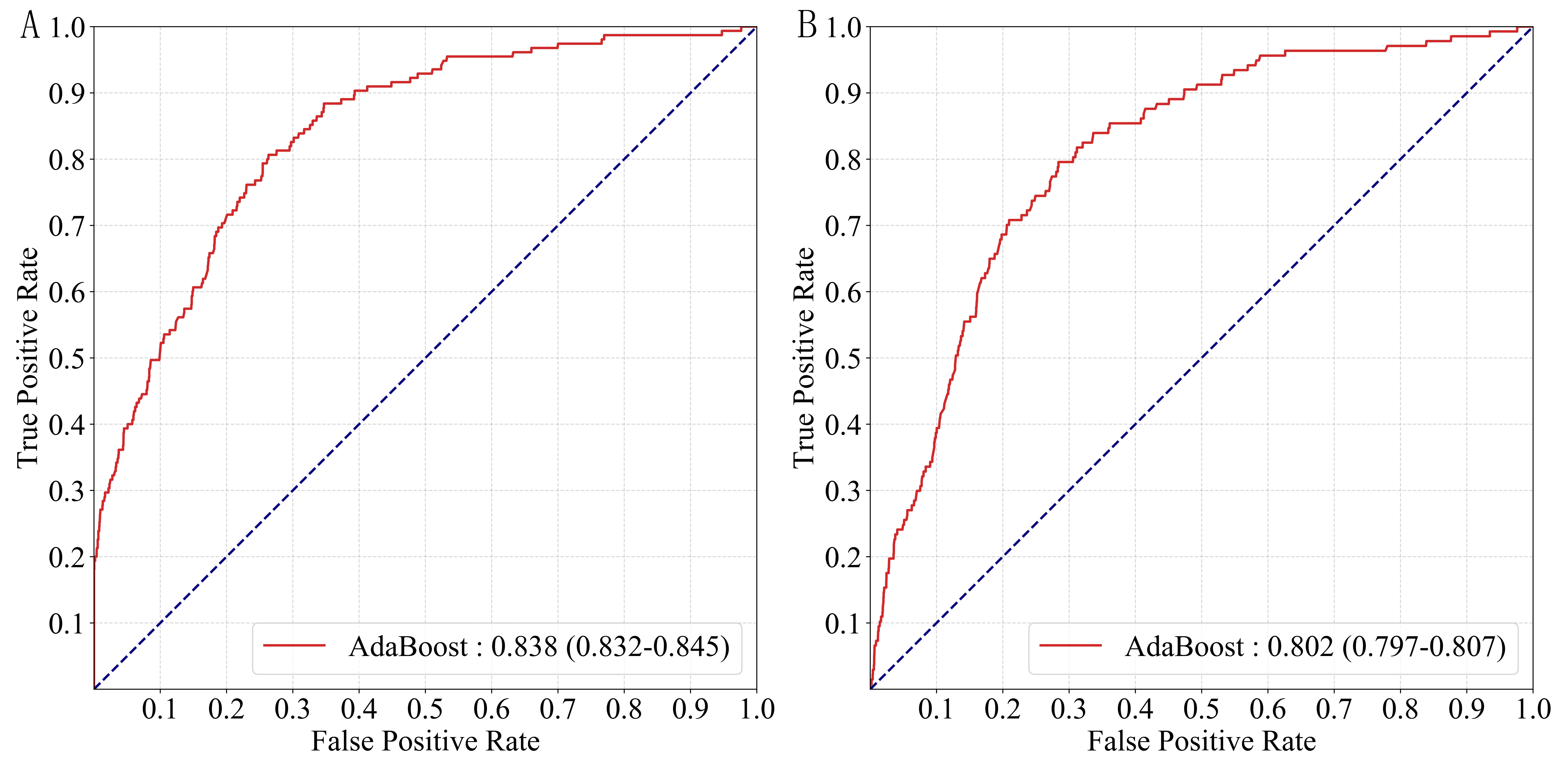

Supplement: Supplementary file 7 — Supplementary Material 7: eFigure 6 AUC curve based on sensitivity analysis of the final selection model. A shows the AUC curve of the model after 6 months of excluding depression. B shows the AUC curve of the model after 2 years of excluding depression [file 13195_2025_1750_MOESM7_ESM.jpg]

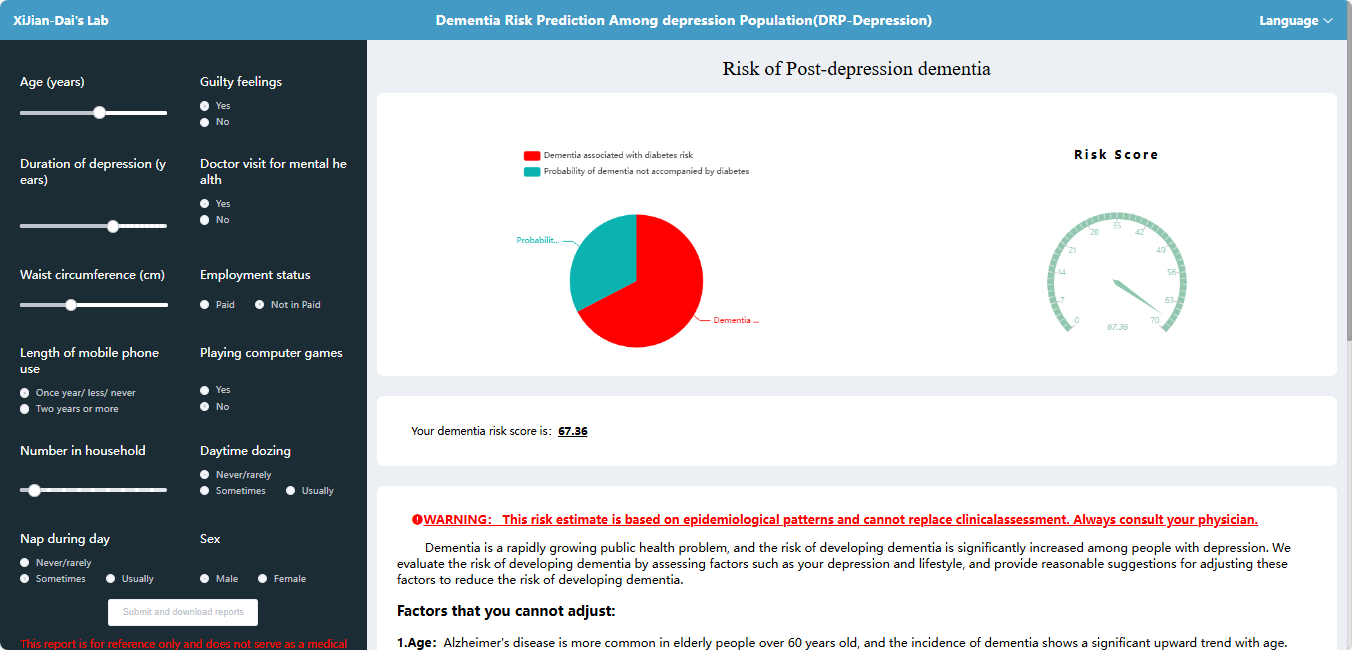

Supplement: Supplementary file 8 — Supplementary Material 8: eFigure 7 Enlarged version of the web interface diagram [file 13195_2025_1750_MOESM8_ESM.jpg]
